# Dissolved Organic Matter in the Coastal Ocean Is Structurally More Diverse Than in Terrestrial Systems, as Shown in an Amazonian Mangrove Estuary

**DOI:** 10.1021/acs.est.5c10721

**Published:** 2026-02-16

**Authors:** Nico Mitschke, Thorsten Dittmar, Michael Seidel

**Affiliations:** † Institute for Chemistry and Biology of the Marine Environment (ICBM), School of Mathematics and Science, 11233 Carl von Ossietzky Universität Oldenburg, Ammerländer Heerstraße 114−118, Oldenburg 26129, Germany; ‡ Helmholtz Institute for Functional Marine Biodiversity (HIFMB) at the Carl von Ossietzky Universität Oldenburg, Oldenburg 26129, Germany

**Keywords:** FT-ICR-MS, high-field NMR, dissolved organic matter, mangroves, dissolved organic sulfur, Amazonia, molecular diversity, sulfurization

## Abstract

Dissolved organic matter (DOM) cycling across the land-ocean continuum is highly complex, and our limited understanding of DOM molecular transformations hinders a full assessment of land-ocean connectivity in the global carbon cycle. Here, we applied one- and two-dimensional high-field ^1^H nuclear magnetic resonance (NMR) spectroscopy and ultrahigh-resolution mass spectrometry (FT-ICR-MS) to investigate sources and transformations of solid-phase extractable DOM along an Amazonian mangrove-fringed river-to-ocean transect. Relative abundances of aromatic compounds decreased from the river to the coastal ocean, whereas aliphatic compounds increased. NMR spectroscopic features, commonly associated with carbohydrates, are probably related to flavonoid- and lignin-derived structural motifs. These structural features were more readily detected by ^1^H NMR spectroscopy, whereas aromatics were more effectively detected by FT-ICR-MS. We tentatively identified polycyclic aromatic sulfur-containing compounds as being predominantly derived from urban areas, whereas sulfurized aliphatic compounds originated from sulfidic mangrove sediments. Surprisingly, while the number of DOM molecular formulas decreased along the river-to-coastal continuum, coastal marine DOM exhibited greater structural diversity than terrigenous DOM. Here, we showed that the interplay of distinct molecular pathways, particularly (photo)­oxidation processes and sulfur incorporation, structurally diversifies DOM in coastal marine environments.

## Graphical abstract

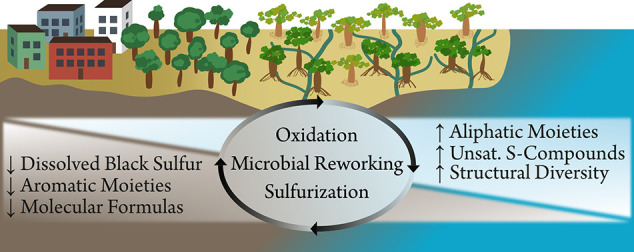

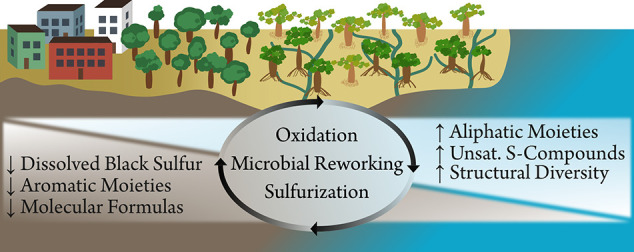

## 1 Introduction

Approximately 0.25 Pg of dissolved organic carbon (DOC) is annually transported by rivers to the ocean,^1^ and about the same amount of DOC enters the ocean from coastal vegetation.^2^ Intertidal ecosystems, such as mangroves, play a crucial role in the delivery of terrestrial dissolved organic matter (DOM) to the ocean. The exchange rate of DOC between mangroves and the ocean strongly depends on the location, but on average, ∼27 g C m^–2^ are directly and ∼202 g C m^–2^ are indirectly (litter that is transformed to DOC) transported to coastal waters.^3^ Thus, mangroves covering a global area of ∼137,760 km^2^ ^4^ are responsible for about 10% of the global flux of DOC from the continents toward the ocean.^5^ Mangroves also play a significant role as carbon sinks in the global carbon cycle.^6^ The second largest area covered by mangroves is in Brazil,^7^ with significant mangrove areas located on the northern coast. Given their important role in carbon sequestration and carbon transport to the ocean, gaining a more profound understanding of the fate of DOM within mangrove-fringed coastal areas is imperative.

Marine DOM is one of the most molecularly diverse mixtures on Earth, consisting of at least hundreds of thousands of distinct organic compounds.^8^ Yet, little is known about its structural and molecular composition, and only a few constituents have been structurally characterized.^9^ One promising approach for the characterization of individual DOM constituents is ultrahigh-resolution mass spectrometry, namely, Fourier-transform ion cyclotron resonance mass spectrometry (FT-ICR-MS). It resolves thousands of molecular masses present in DOM, yet it does not resolve isomers and yields only limited structural information. In contrast, nuclear magnetic resonance (NMR) spectroscopy is an analytical technique, giving structural insights into organic compounds on an atomic level. Recent technical and methodological improvements have made the application of NMR spectroscopy for the analysis of DOM more feasible,^10^ revealing different structural information and thus complementing the results obtained by FT-ICR-MS.^11,12^

DOM in mangrove-fringed estuaries is derived from terrestrial plants, mangrove trees, and microbial and algal sources, and its highly complex cycling is still not well understood. In the present study, we applied complementary FT-ICR-MS and one-dimensional (1D) ^1^H as well as two-dimensional (2D) high-field NMR spectroscopy, in particular ^1^H,^1^H correlation spectroscopy (COSY), for the compositional analysis of DOM from a mangrove-fringed river-to-coastal ocean transect. In mangrove-fringed coastal systems, a multitude of spatially heterogeneous processes shape the molecular composition of DOM. In the open ocean, DOM from various coastal sources mixes with recalcitrant marine DOM, which has been reworked by marine microorganisms over time scales ranging from years to millennia. We hypothesize that marine DOM exhibits greater structural diversity than terrigenous DOM due to its diverse sources and complex microbial and abiotic transformations. Furthermore, we propose that this trend in structural diversity has been overlooked so far because it is obscured at the molecular formula level and only emerges through complementary structural analytical approaches. We suggest that the complementary application of FT-ICR-MS and high-field NMR spectroscopy identifies source-specific molecular fingerprints, providing detailed insights into the source distribution of DOM and molecular transformation processes at the land-ocean interface.

## 2 Materials and Methods

### 2.1 Study Site and Sampling

The study site is in the Amazonian coastal zone in North Brazil. For this study, samples at 23 stations along a riverine transect of the Caeté River (Pará, Brazil) and low-tide samples from the Furo do Meio tidal creek were considered [S1.1, Supporting Information (SI)]. These samples were pooled into eight samples, which were analyzed by FT-ICR-MS and NMR spectroscopy. Original samples were taken between 27 September 2017 and 10 October 2017 and further processed as described previously.^13^ Briefly, samples were filtered through 1.0 μm Causapure filter cartridges (CPR-001–09-DOX, Infiltec GmbH), acidified with diluted hydrochloric acid (∼8 mol L^–1^) to pH 2 and solid-phase extracted (SPE, Bond Elut PPL, Agilent Technologies Inc.), eluting the cartridges with methanol after desalination as described by Dittmar et al.^14^ In our combined samples, the SPE efficiency was higher in terrestrial samples (Table S1), either because they contain more SPE-extractable DOM or due to salinity effects. Since DOM extraction efficiencies of PPL cartridges increase with salinity,^15^ the higher efficiencies in low-salinity terrestrial samples likely reflect a greater abundance of SPE-accessible DOM. Selected environmental parameters and metadata are summarized in Table S1 (SI). Unless otherwise noted, all discussed trends, including references to sample origins (e.g., river or ocean), refer to SPE-DOM.

### 2.2 FT-ICR-MS

FT-ICR-MS data were reused from Knoke et al.^13^ and are publicly available on PANGAEA.^16^ Molecular characterization by ultrahigh-resolution mass spectrometry was done using a solariX XR FT-ICR-MS (Bruker Daltonics GmbH) equipped with a 15 T magnet (Bruker BioSpin GmbH) and an Apollo II electrospray ionization source (Bruker Daltonics GmbH), operated in negative ionization mode. For comparison with the NMR measurements, FT-ICR-MS data of pooled samples were generated by averaging the data of individual samples. The applied analytical conditions and further processing steps of the MS data, including molecular formula attribution, are described in more detail in the SI (S1.3). Selected intensity-weighted molecular parameters are summarized in Table S2 (SI).

### 2.3 NMR Spectroscopy

Since the amounts of individual samples would have been too low for NMR analysis (∼1.0–2.0 mg DOC), 23 samples were pooled to yield eight samples, each corresponding to 5 mg SPE-DOC. The samples were designated as RW (river water sample), BW1–BW4 (brackish water samples from the mangrove-fringed section), SW (coastal ocean seawater sample), NT (low tide sample collected during neap tide), and ST (spring tide sample collected during neap tide), respectively (Figure S1, SI). Methanol was evaporated from the aliquots under a stream of argon at 40 °C, samples were redissolved in 100 μL of methanol-*d*_4_ (CD_3_OD) (99.95 atom % D, MagniSolv, Merck KGaA) and the solvent was evaporated as described before. Three cycles of evaporating and redissolving were applied to remove traces of nondeuterated methanol and to ensure complete exchange of exchangeable protons with deuterium. The samples were finally dissolved in 600 μL of CD_3_OD and transferred into 5 mm NMR tubes. Measurements were performed at Bruker BioSpin’s user facility in Ettlingen (Germany) on a Bruker AVANCE NEO 800 MHz instrument (Bruker Biospin GmbH) with a cryogenic 5 mm TCI probe, and basic processing was conducted with TopSpin (versions 4.1.4 and 4.3.0, Bruker BioSpin GmbH) as described in the SI (S1.5). Processed 1D ^1^H NMR spectra are displayed in the SI (S2.1). Further data processing was done in MATLAB (version R2022b, The MathWorks, Inc.) using in-house-written scripts (S1.6, SI). For ^1^H NMR data the essential steps were: (1) data point transformation (Figure S2, SI), (2) removal of solvent regions (4.70–5.05 ppm for H_2_O and 3.20–3.45 ppm for CH_3_OH), (3) integration of sections corresponding to specific structural features (Figure S4, SI), (4) defining a subregion of interest for further analysis, (5) binning, (6) normalization, (7) generation of a data matrix with the samples as rows and the intensity of each bin as columns. Bins without signal intensity throughout all samples were removed prior to further analysis. COSY NMR data were processed analogously with the additional step of noise removal from the raw spectra using a signal-to-noise ratio (SNR) cutoff of 3, whereas the exclusion of solvent signals was not necessary. COSY spectra with a bin size of 0.05 ppm, which were used for further analysis, are displayed in the SI (Figure S3). For both types of experiments, only the region between 0 and 10 ppm was analyzed. NMR spectral predictions for individual substances were performed as described in S1.7 (SI).

### 2.4 Optimal Bin Size for NMR Data

The signal positions in NMR spectroscopy (chemical shifts) depend on the temperature and sample matrix (concentration, pH, ionic strength, etc.). The effects of this variability in further analyses can be reduced by postprocessing, such as spectral alignment or binning. The simplest binning approach divides the spectrum into equally spaced sections with defined chemical shift widths. While binning may help to compensate for chemical shift fluctuations, it also reduces spectral information. To determine the number of bins representing the best compromise (“trade-off”) between the compensation of chemical shift fluctuations and the preservation of information, we calculated the average Bray–Curtis dissimilarity among all samples for bin sizes between 0.01 and 1 ppm. In the plot of Bray–Curtis dissimilarities versus the resulting number of bins (Figure 1), we considered the knee point (determined in MATLAB with *knee_pt* by Dmitry Kaplan, version 1.1.0.0) as the best compromise between bin size and information content. Optimal bin sizes were determined to be 0.10 ppm for 1D ^1^H NMR spectra and 0.05 ppm for 2D COSY NMR spectra. This is almost consistent with the frequently used bin size of ∼0.05 ppm for 1D ^1^H NMR spectra in metabolomic studies.^17^

**Figure 1:**
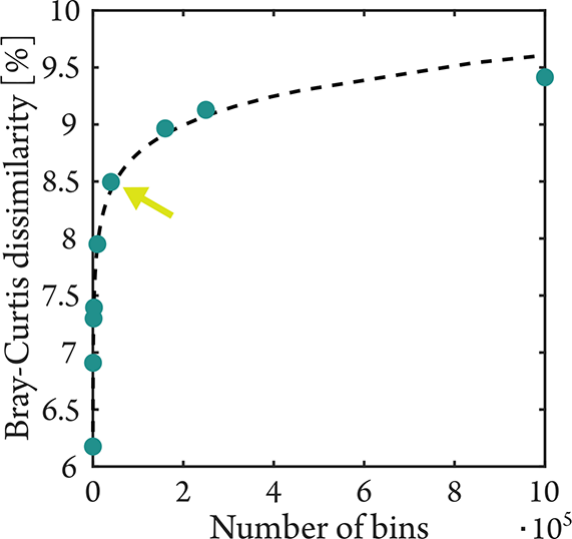
Average Bray–Curtis dissimilarity among all samples of COSY data as a function of the number of bins, demonstrating a logarithmic relationship (dotted line, *R* ^2^ = 0.9933). The knee point was determined to be at 40,000 bins (arrow), corresponding to a bin size of 0.05 ppm.

### 2.5 Structural Group Classification in NMR Spectra

Signals of properly acquired 1D ^1^H NMR spectra are fully quantitative, and conclusions about the abundances of structural features on a proton basis can be drawn by integration of spectral sections. Commonly used sections represent aromatic, olefinic, carbohydrate and methoxy, acetyl, and aliphatic structural motifs. The exact classifications and chemical shift values may vary.^12,18,19^ We used the classification scheme adapted and modified from Hertkorn et al.^12^ (SI, Figure S4, section integral values in Table 1). Modifications include slight differences in the chemical shift boundaries of sections and renaming of certain sections. The sections referred to as “carboxyl-rich alicyclic molecules”^20^ (CRAM), “acetate-analogues” as well as “carbohydrate-like and methoxy” are herein referred to as “protons in the α-position to carbonyls”, “acetyl protons”, and “protons in the α-position to single-bonded oxygen”, respectively, being more general descriptions for the majority of DOM structural features expected to cause signals in these sections. Please note that the section labels intentionally exclude nitrogen-, sulfur-, and phosphorus-containing structural motifs, as these heteroatoms occur at much lower abundances in DOM than oxygen.

**Table 1:** Relative Abundances (in %) of Hydrogen in Key Structural Features Determined by Integration of the Respective Sections from 1D ^1^H NMR

structural features	aliphatic protons^a^	acetyl protons^a^	protons α to carbonyl^a^ ^,^ [Table-fn t1fn3]	protons α to oxygen^b^ ^,^ [Table-fn t1fn4]	olefinic protons^b^	aromatic protons^b^
δ_H_ (ppm)	0.0–1.9	1.9–2.3	2.3–3.0	3.0–5.3	5.3–6.5	6.5–10.0
seawater (SW)	37.2	11.1	18.0	27.0	2.4	4.3
brackish water (BW4)	34.1	10.7	17.5	29.3	3.0	5.4
brackish water (BW3)	35.0	11.0	18.0	28.9	1.7	5.3
brackish water (BW2)	34.9	11.1	17.9	28.9	2.1	5.2
brackish water (BW1)	30.4	9.8	15.9	30.3	4.2	9.4
river water (RW)	30.0	9.3	15.4	31.6	4.5	9.1
neap tide (NT)	30.3	10.2	18.6	31.0	2.8	7.0
spring tide (ST)	34.6	10.9	18.9	27.5	1.4	6.7

* Increasing abundances along the river-to-ocean transect.

# Decreasing abundances along the river-to-ocean transect.

a Protons in the α-position to carbonyls.

b Protons in the α-position to single-bonded oxygen.

More detailed structural classifications can be obtained from 2D NMR experiments. A classification scheme of COSY spectra for DOM analysis has been proposed by Hertkorn et al.^12^ and later modified by Seidel et al.^11^ Here, we revised this classification, defining 19 sections corresponding to distinct structural features (Figure 2, for more detailed explanations, see S1.9, SI). Please note that the COSY experiments performed in the present study are not quantitative. The intensity of a COSY cross-peak is influenced not only by the compound concentration but also by physical parameters such as *J*-coupling constants and relaxation times (T1 and T2). In complex mixtures like DOM, individual spectral regions may contain overlapping signals from multiple chemical motifs, and subtle structural variations can affect cross-peak intensities independently of concentration. Because all samples were collected along a single river-to-ocean continuum, we do not expect entirely unrelated compounds to dominate the same spectral regions. Under this assumption, we use COSY intensities of equal spectral regions in a semiquantitative, trend-focused manner for correlation analysis. Relative abundances of different structural motifs within a single sample should also not be interpreted quantitatively. Both, 1D and 2D NMR sections, are based on idealized, fixed chemical shift ranges. Consequently, signals corresponding to a specific structural motif may, to some extent, be operationally assigned to adjacent sections. For example, certain olefinic protons might be detected as aromatic protons and *vice versa*.

**Figure 2:**
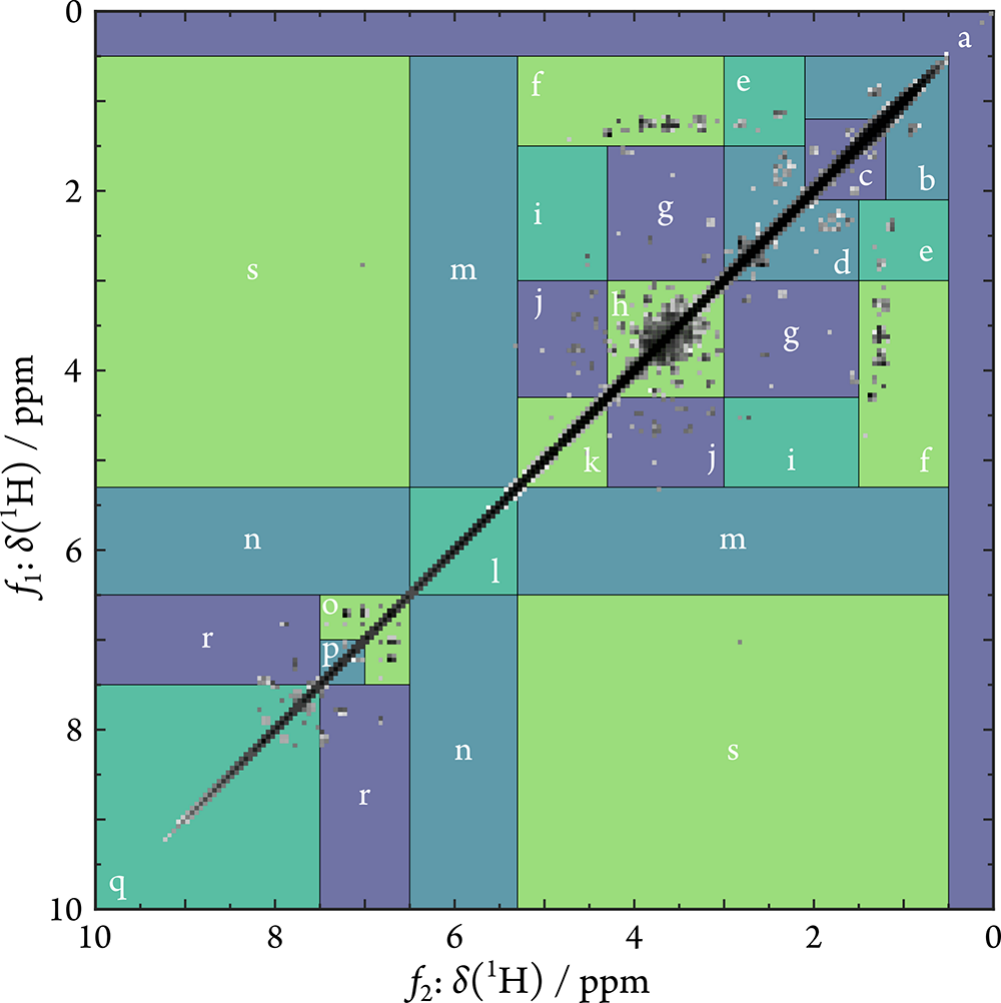
Binned COSY NMR spectrum (800 MHz, CD_3_OD, bin size 0.05 ppm) of the seawater sample and division into 19 sections representing protons of distinct structural motifs. Signals are indicative for (a) cyclopropyl motifs; (b) pure aliphatic methyl groups; (c) aliphatic CH- and CH_2_-connections (“intra-aliphatic”); (d) benzylic-, *N*/*S*-, and carbonyl-adjacent alkyl groups (alkyl > ethyl); (e) benzylic-, *N*/*S*-, and carbonyl-adjacent ethyl and 1-methylalkyl groups; (f) ethoxy- and 1-methylalkoxy groups, (g) alkoxy groups (alkyl > ethyl); (h) vicinal diols and their derivatives; (i) 2-deoxy acetals; (j) anomeric protons of common saccharides; (k) acetal protons (self-correlation); (l) olefins; (m) olefins attached to (modified) aliphatic motifs; (n) aromatics heavily substituted with electron-donating groups (EDGs), electron-withdrawing groups (EWGs), and/or heteroatoms, occasionally olefins attached to aromatics (^4^ *J* correlations); (o) electron-rich aromatics; (p) pure aromatics; (q) polycyclic aromatic compounds (PACs), electron-poor aromatics; (r) electron-rich PACs, aromatics substituted with EDG and EWG; and (s) biomolecules, aldehydes, occasionally substituted aromatics (^4^ *J* correlations).

It is important to note that off-diagonal peaks in COSY always represent the coupling between two protons. Thus, signals in a given section, such as section “a” may not only correspond to cyclopropyl-derived protons but also to protons that are attached to cyclopropyl protons *via* most likely three bonds. Thus, we prefer to use the term “structural motifs” when referring to structural information derived from 2D NMR, rather than attributing signals to specific protons.

### 2.6 Multivariate Statistics

Multivariate statistical analyses were conducted in R Studio (version 2023.06.0; R version 4.3.1) using Bray–Curtis dissimilarities among samples calculated from binned NMR data matrices and normalized signal intensities of molecular formulas from FT-ICR-MS data. Principal coordinate analyses^21^ (PCoA) were conducted on the Bray–Curtis dissimilarity matrices to assess the variance among samples. The first two PCs were considered for further analyses. The relative abundances of protons from specific structural moieties (from 1D ^1^H and 2D COSY), intensity-weighted molecular groups and parameters (from FT-ICR-MS), environmental parameters, and metadata were fitted *post hoc* to the PCoA scores using the *envfit* function as implemented in the *vegan* ^22^ package (version 2.6–4). Correlations of parameters with PCoA scores were tested with 9,999 permutations and were considered significant if *p* < 0.05 or *p* < 0.10. Occasionally, parameters with *p* < 0.15 were displayed in the PCoA plots for data discussion. Correlations with one of the ordinations were considered strong when *r* > 0.50. Spearman correlations were calculated and considered for discussion if *p* ≤ 0.1. The data sets obtained by NMR spectroscopy and MS analyses were linked by canonical correlation analysis^23^ (CCorA) as described by Osterholz et al.^24^ The first two PCs from the NMR- and MS-based PCoAs were used. The significance of canonical correlations was tested with 719 permutations, and the permutational probability associated with Pillai’s trace was 0.04.

## 3 Results and Discussion

### 3.1 ^1^H NMR and FT-ICR-MS Analyses Reflect Comparable Trends but Suggest Different Relative Abundances for Aliphatic and Aromatic DOM Compounds

The quantitative 1D ^1^H NMR data showed that, on a hydrogen basis, aliphatic structural moieties were least abundant in the river sample (30%) and increased with increasing salinity to 37% in the seawater sample (Table 1). The only exception to this salinity-dependent trend occurred in the tidal creek at neap tide, where the proportion of aliphatic protons was only 30%. Similar trends were also observed for the unsaturated and highly unsaturated compound groups derived from FT-ICR-MS data (Table 2), which also include compounds with aliphatic structural moieties. Unsaturated compounds increased from 8 to 10%, while highly unsaturated compounds increased from 65 to 78% from low to high salinity. Aliphatic moieties (NMR) increased by a factor of 1.24, while unsaturated and highly unsaturated compounds (FT-ICR-MS) increased by 1.29 and 1.18, respectively, thus indicating a similar trend for both analytical methods. An increasing abundance of aliphatic structures with increasing salinity has been reported before,^5^ likely reflecting marine microbial inputs^25^ and fewer aromatic compounds from vascular land plants, which are preferentially removed by photodegradation^5,26–28^ and coprecipitation with metals.^29^

**Table 2:** Relative Abundances (in %) of Compound Groups as Determined by FT-ICR-MS Analysis[Table-fn t2fn3]

sample	saturated	unsaturated^a^	highly unsaturated^a^	aromatics[Table-fn t2fn2]	PACs[Table-fn t2fn2]	saccharides
seawater (SW)	0.16	10.1	77.8	8.3	2.1	0.09
brackish water (BW4)	0.12	10.0	71.2	11.9	4.7	0.14
brackish water (BW3)	0.18	9.7	68.9	13.4	5.6	0.11
brackish water (BW2)	0.13	9.3	67.8	14.3	6.2	0.14
brackish water (BW1)	0.11	7.9	68.4	14.8	6.6	0.11
river water (RW)	0.42	7.8	65.4	15.8	8.2	0.25
neap tide (NT)	0.20	7.5	70.7	14.4	4.9	0.02
spring tide (ST)	0.04	7.0	75.1	12.8	3.0	0.00

* Increasing abundances along the river-to-ocean transect.

# Decreasing abundances along the river-to-ocean transect.

a Saturated compounds: DBE = 0, unsaturated compounds: 2.0 ≥ H/C ≥ 1.5, highly unsaturated compounds: AI_mod_ ≤ 0.50 and H/C < 1.5, aromatics: 0.67 ≥ AI_mod_ > 0.5, PACs: AI_mod_ > 0.67, saccharides: O/C ≥ 0.7, 2.2 > H/C ≥ 1.7.

In addition, the hydraulic gradient, i.e., the pressure difference created by changing water levels between high and low tide, drives porewater discharge into tidal creeks during low tide.^30^ Accordingly, porewater likely dominates the molecular composition of DOM at low tide. Consistent with this expectation, we observed more highly unsaturated compounds by MS (Table 2), characteristic of porewater-derived DOM.^13,31^ NMR also revealed an increased abundance of protons in the α-position to carbonyls (Table 1), indicative of CRAM,^20^ carboxyl-rich alicyclic molecules associated with unsaturation, which aligns with the increased abundance of highly unsaturated compounds as detected by MS.

Consistent with terrestrial inputs,^32^ aromatic and olefinic protons as detected by NMR analysis were more abundant in samples with lower salinity (9 and 4%, respectively) compared to samples with higher salinity (5 and 3%, respectively). Similarly, MS-derived aromatics (sum of aromatics and polycyclic aromatic compounds [PACs]) decreased from 24 to 10% along the transect. Both methods thus captured a comparable decline in aromatic compounds (MS) and aromatic or olefinic moieties (NMR) from the river to the ocean.

Differences between MS and NMR largely arise because FT-ICR-MS detects entire compounds and yields molecular formulas, while NMR detects structural motifs. A single compound, containing several structural features, yields one molecular formula but multiple NMR signals. In addition, FT-ICR-MS is affected by ionization bias and is not strictly quantitative,^33^ while ^1^H NMR underrepresents hydrogen-poor structures, such as aromatics. This is evident when comparing aromatic proportions: FT-ICR-MS detects more aromatics than NMR because aromatic carbons often lack protons. In addition, the MS-based classification as “aromatics” is inferred from double bond equivalent (DBE)-based indices, specifically the modified aromaticity index^46,47^ (AI_mod_). Consequently, compounds with high unsaturation from ring closures and/or double-bonded heteroatoms can be assigned as “aromatics” even when true aromatic rings are less prominent. Together, these factors can lead to an overrepresentation of aromatics in MS-derived groups. Likewise, compounds are classified based on unsaturation (H/C < 1.5, AI_mod_ ≤ 0.5) as “highly unsaturated” based on FT-ICR-MS. Thus, oxygen-rich alicyclic compounds, including CRAM-like structures,^20^ may fall into the “highly unsaturated” group while contributing little or no olefinic/aromatic signal in ^1^H NMR. MS compound groups should therefore be treated as descriptive classifications rather than as quantitative structural proxies. Despite these methodological differences, both techniques revealed consistent trends along the transect, underscoring the robustness of the results. The observed correlations do not imply, however, that identical compounds were responsible but rather that molecular and structural features covaried spatially, reflecting similar biogeochemical processes, sources, and sinks.

### 3.2 Oxygen-Containing Structural Motifs Are Further Oxidized Along the River-to-Ocean Continuum

The combined abundance of oxygen-associated structural features (acetyl protons, protons in the α-position to carbonyls, and protons in the α-position to single-bonded oxygen) varied only by up to 4% along the river-to-ocean transect (Table 1). While this suggests that the overall oxygen content in DOM compounds remained relatively stable, individual structural motifs varied by up to 19% (Table 1), indicating dynamic shifts in the mode of oxygen incorporation into DOM compounds. Specifically, single-bonded oxygen species (protons in the α-position to single-bonded oxygen) decreased along the transect, whereas double-bonded oxygen species (acetyl protons and protons in the α-position to carbonyls) increased correspondingly. The increase in structural features containing double-bonded oxygen (4.4%) nearly compensated for the decrease in single-bonded oxygen species (4.6%), pointing to redistribution of oxygen functionalities rather than a net gain or loss.

The relative stability of average O/C values in molecular formulas (ranging from 0.38 to 0.39) further supports a constant oxygenation state of DOM compounds across the transect. While this stability could theoretically result from the complete removal of low-oxidized DOM compounds and simultaneous production or release of highly oxidized species, such a coincidentally precise balance is unlikely. Instead of a net loss of low-oxidized DOM and concurrent production of highly oxidized species, our results indicate transformations of low-oxidized moieties (e.g., alcohols) into higher-oxidized forms (e.g., ketones) while preserving the core carbon skeletons of DOM compounds. A dominant input of highly oxidized compounds without a corresponding sink would have resulted in elevated O/C ratios, which was not observed. Photooxidation and biodegradation are likely drivers of these transformations.^34^ For example, photooxidation alters oxygen functionalities in DOM in oligotrophic marine systems^35^ and causes partial oxidation of terrestrial DOM in Arctic surface waters.^36^

### 3.3 Opposing Trends of DOM Molecular Formula and Structural Diversity

After processing, a total of 2,226 signal bins were included in the COSY NMR analysis. In seawater DOM, 1,302 bins with a signal intensity greater than zero were detected, whereas only 624 bins with signals were detected in riverine DOM (Figure 3A; S2.2, SI). The highest number of bins with signals were found in neap (1,754) and spring tide DOM (1,925). Thus, seawater and tidal creek samples appeared structurally more complex than fluvial and brackish water samples, a difference clearly visible in the COSY spectra (Figure S3, SI).

**Figure 3:**
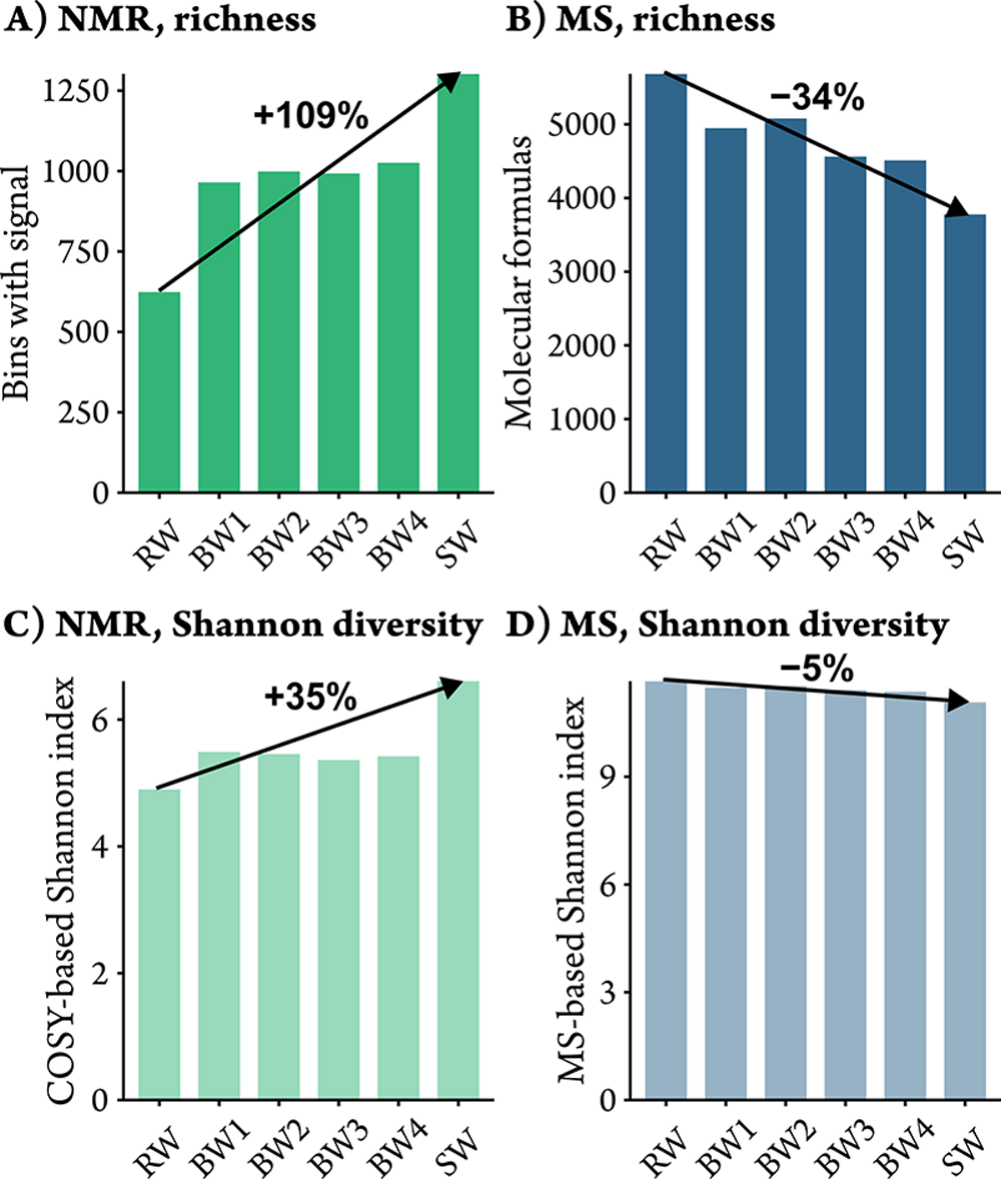
(A) Number of detected bins with signal intensity, (B) number of molecular formulas, (C) Shannon diverstiy calculated from cross-peak bin intensities (excluding the diagonal, *cf*. S2.3, SI), and (D) Shannon diverstiy calculated from molecular formula intensities for the six river-to-coastal ocean samples. The diagonal was excluded from the COSY NMR-based Shannon diverstiy calculation, because up to 99.7% of the spectral intensity is concentrated along the diagonal (*cf*. S2.4, SI), which would otherwise skew the results. The Shannon index was calculated using a base-2 logarithm.

In total, 8,686 distinct molecular formulas were identified by FT-ICR-MS analysis. In the transect samples, most molecular formulas (5,684) were detected in river water (S2.2, SI), decreasing along the transect to 3,778 in the seawater (Figure 3B). In the tidal creek, 6,118 and 6,896 molecular formulas were detected at spring and neap tide, respectively. Thus, DOM was richest in terms of structural features (NMR) and molecular formulas (MS) at low tide in the creek. Interestingly, opposite trends in structural and molecular formula diversity were observed along the transect: riverine DOM contained more molecular formulas but fewer structural features than seawater DOM. This suggests that, despite greater compositional richness in terms of molecular formulas, riverine DOM is structurally less diverse or contains fewer NMR-detectable isomers. This is also evident from the Shannon diversity index^21^ calculated from bin intensities of cross-peaks (excluding the diagonal, *cf*. S2.3, SI) and from molecular formula intensities (Figure 3C,D). The Shannon diversity index captures diversity by considering both evenness and richness. These results indicate that structural diversity is hidden at the molecular formula level but revealed through 2D NMR analysis.

Oceanic DOM^37^ is typically older than riverine DOM,^2^ suggesting that it has undergone more extensive transformation processes. During these transformations, DOM loses source-specific compounds (decreasing β-diversity) and gains more universal compounds (increasing α-diversity).^38^ Consistent with this concept, marine DOM was structurally more diverse than riverine DOM when analyzed by NMR, likely due to the mixing of terrestrial and coastal inputs and further microbial and abiotic processing. This also implies that marine DOM likely contained more isomers per molecular formula, as the lowest number of molecular formulas was found in the coastal ocean DOM.

An alternative explanation is that the riverine DOM contained numerous structural isomers with nonoverlapping (i.e., nonadditive) signals in 2D NMR. In that case, their low signal intensities may be below the detection limit of comparatively insensitive NMR analysis. However, this seems unlikely as tidal creek DOM showed high diversity in both NMR and FT-ICR-MS, suggesting that detectability alone cannot explain the observed pattern.

### 3.4 Structural Diversity Exceeds Molecular Formula Diversity

At first glance, DOM samples appeared more dissimilar at the molecular formula level (FT-ICR-MS) than at the structural level (COSY NMR), based on Bray–Curtis dissimilarity. Molecular formula dissimilarity ranged from 7–36% (Figure S15C, SI), while structural dissimilarity was 2–15% (Figure S15A, SI). However, COSY diagonal peaks store most of the spectral intensity but only little structural information. Including them in the dissimilarity analysis may inflate the similarity and obscure meaningful differences in cross-peaks, which are more indicative of structural dissimilarities.

To test this hypothesis, we removed the diagonal signals (S2.3, SI) and reanalyzed the data. Structural dissimilarities then increased to 11–87% (Figure S15B, SI), suggesting that various structural motifs occurred within compounds sharing the same molecular formula. This finding aligns with a previous open ocean study demonstrating greater structural (32–68%) than molecular formula (9–25%) dissimilarity.^11^ Notably, diagonal peaks were also ignored in this study.

The observed structural and compositional differences in DOM across the transect were further supported by PCoA analyses, revealing clear gradients from fluvial to marine DOM and from mangrove-influenced to low-tide samples. Both COSY-based (Figure 4A,B) and MS-based (Figure 4C,D) PCoAs revealed similar patterns: PC1 distinguished transect and low-tide samples, while PC2 separated fluvial from marine samples. This was also reflected in the correlations of the environmental parameters and MS- or fluorescence-derived indices (Figure 4B,D) with the ordinations. Because all SPE-DOM extracts were adjusted to identical SPE-DOC concentrations prior to NMR and FT-ICR-MS analyses, the observed correlations with DOC (Figure 4B,D) cannot be attributed to concentration effects. Rather, they reflect compositional differences along the transect, where variations in DOM sources and transformation processes are mirrored in the molecular composition of the SPE-DOM fractions. PC1 correlated positively with the index for sulfurized DOM from porewater (*I*_SuP_),^13^ reflecting mangrove porewater input. PC2 correlated positively with salinity, δ^13^C SPE-DOM values, and the biological index (BIX),^39^ a proxy for biologically modified DOM, and negatively with the humification index (HIX),^40^ an indicator for the degree of DOM humification. Notably, 1D ^1^H NMR (red vectors in Figure 4A) captured structural motif changes along the transect, whereas COSY-derived structural groups (ocher vectors in Figure 4A) were required to distinguish the transect from the low-tide samples.

**Figure 4:**
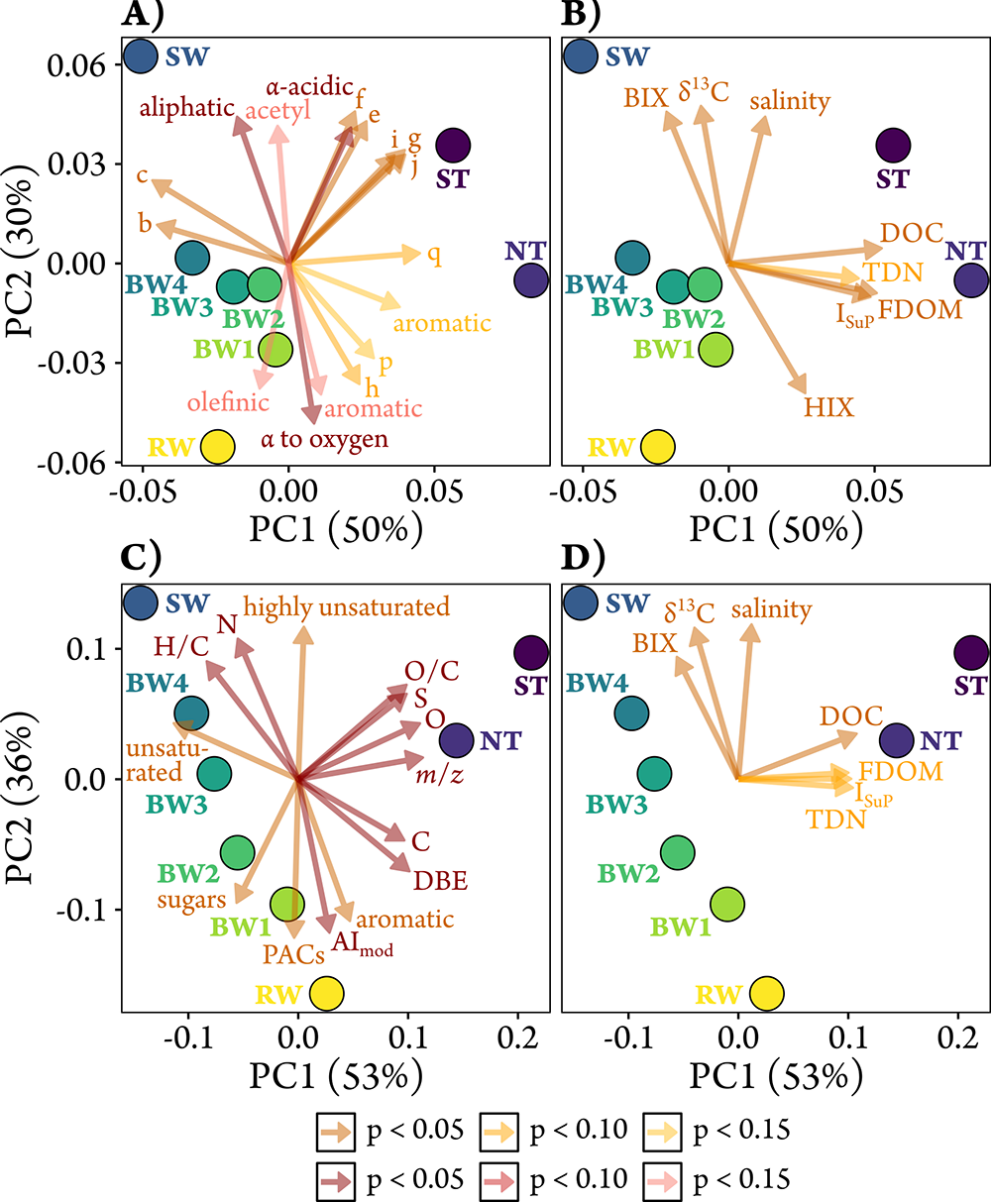
Principal coordinate analyses (PCoA) based on Bray–Curtis dissimilarity matrices of binned COSY NMR data (A, B) and FT-ICR-MS data (C, D). Correlations with relative abundances of structural feature groups derived from 1D ^1^H (red vectors) and 2D COSY (ocher vectors) NMR spectra (A), environmental, sample, and FT-ICR-MS parameters (B, D) as well as FT-ICR-MS-derived intensity-weighted molecular parameters (red vectors) and groups (ocher vectors) (C) were fitted *post hoc* to the PCoA of NMR and FT-ICR-MS data, respectively. Please note that the ocher vector representing aromatic structural motifs derived from 2D COSY reflects the combined contributions of all aromatic regions. Correlations with *p* < 0.10 (standard colors) and with *p* < 0.05 (dark colors) are shown. Occasionally, correlations with *p* < 0.15 are displayed (light colors). The projections of sampling points onto vectors have maximum correlation with the respective variables.

The first two principal coordinate axes of the COSY data-based PCoA explained 80% of structural variability compared to 89% of the molecular formula variability explained by the MS data-based PCoA. This difference is consistent with the dissimilarity analysis, as NMR captures a broader array of DOM structural features, making them harder to condense into just two axes.

FT-ICR-MS resolves thousands of distinct mass peaks and excels at detecting discrete molecular signatures of specific DOM sources, such as terrestrial^41^ or porewater-derived^13^ DOM. Even small changes in DOC concentrations caused by a changing relative abundance of only a few individual compounds can likely be detected, but such subtle changes are unlikely to be captured by NMR. In contrast, NMR more easily detects bulk compositional changes in functional groups, such as those resulting from photooxidation or microbial transformation,^19,20,35^ which may remain obscured at the molecular formula level. This makes FT-ICR-MS especially powerful for tracing processes involving a few dominant key components, while NMR is more sensitive to functional group transformations affecting bulk DOM. Combined, the two methods offer complementary insights. FT-ICR-MS reveals detailed source-specific molecular composition, while NMR captures structural transformations, together providing a more comprehensive understanding of DOM dynamics essential for modeling carbon cycling and predicting ecosystem responses.

### 3.5 Lignin-Derived Compounds in Terrigenous DOM Yield Carbohydrate-Like Structural Signals

Although SPE with PPL cartridges is known to yield low recoveries of small, highly polar compounds such as saccharides,^42^ the 1D ^1^H NMR region representative for protons in the α-position to oxygen is often at least partly attributed to carbohydrate structural motifs.^12,43,44^ It is therefore notable that the abundance of anomeric protons (j), indicative of saccharides, was not correlated with vicinal diols (h) (*cf*. vectors “j” and “h” in Figure 4A). This suggests either that most vicinal diol signals (h) were not carbohydrate-derived or that many anomeric protons were not part of carbohydrate-like structures. Because the anomeric proton region (j) (especially 4.6–5.0 ppm) is highly specific for saccharides, the former explanation appears more likely.

To examine this further, we reintegrated all COSY sections after removing diagonal signals (Figure S13, SI) and re-evaluated their correlations with the PCoA ordinations based on binned COSY data without diagonal removal (Figure S14B, SI). In this analysis, vicinal diols (h) correlated with anomeric protons (j), acetal (i), and *O*-alkyl motifs (g). This indicates that cross-peaks in the vicinal diol section (h) are indeed related to saccharide-like structures, likely existing in deoxygenated forms, such as 3-deoxy sugars (to explain correlations to *O*-alkyl moieties). Such deoxygenated sugars have been identified in marine high-molecular-weight DOM.^45^

Also, the 1D NMR section representing protons in the α-position to single-bonded oxygen was almost uncorrelated with the carbohydrate-related COSY sections (i, g, j), suggesting that the predominant signal intensity of the quantitative 1D NMR section originates from noncarbohydrate structures. Thus, much of the diagonal signal intensity of the vicinal diol section (h) is likely caused by protons with almost no coupling to other protons over three bonds or by protons that couple only to chemically similar protons, with cross-peaks appearing close to or on the diagonal. This indicates the presence of methoxy groups (e.g., in ethers or methyl esters) or 1,3-dicarbonyls. These structures contain electron-withdrawing motifs that would cause the observed characteristic low-field shift (∼3.0–4.3 ppm), resulting in spectral overlap with carbohydrate signals. Note that this overlap does not imply a biogeochemical relationship. Other structural motifs, such as alkyl halides, may also contribute signals in this region, but they are unlikely to be major DOM constituents.

Further evidence for noncarbohydrate contributions to the 1D section representing protons in the α-position to single-bonded oxygen was obtained from correlations between 1D NMR section integrals and molecular formulas in van Krevelen space (Figure 5). Molecular formulas with high O/C (>0.4) and H/C (>1.0) values, typical for carbohydrates, were negatively correlated with protons in the α-position to single-bonded oxygen (Figure 5A). In contrast, molecular formulas with lower H/C and O/C values, i.e., more aromatic compounds, were positively correlated. However, other 1D NMR sections exhibited expected correlations: molecular formulas with high H/C values (>1.0) were positively correlated with the aliphatic 1D section (Figure 5B), and compounds with low H/C values (<1.0) were positively correlated with the aromatic 1D section (Figure 5C). Interestingly, aromatic protons also correlated with formulas of relatively high H/C (>0.7) and O/C values (Figure 5C, red polygon), often not classified as aromatic by molecular formula indices such as the AI_mod_ (*cf*. red circled dots in Figure 5C).^46,47^ This suggests that molecular formula indices alone may underestimate aromaticity, probably because FT-ICR-MS detects entire compounds, while NMR resolves structural motifs (*cf*. discussion below).

**Figure 5:**
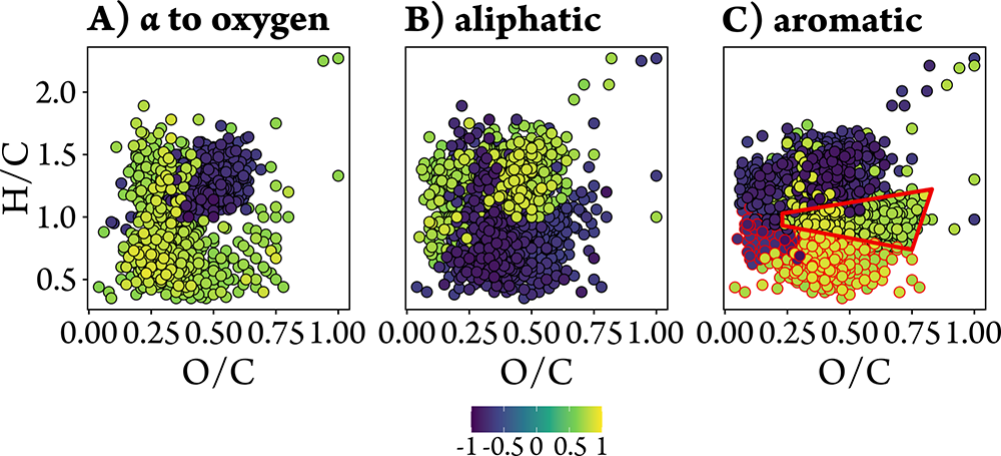
Van Krevelen diagrams displaying molecular formulas with positive (green to yellow) and negative (blue) Spearman correlations of molecular formula abundances with (A) protons in the α-position to single-bonded oxygens, (B) aliphatic protons, and (C) aromatic protons. Only molecular formulas with significant correlations (*p* < 0.1) are shown, colored by the correlation coefficient (*r*) and layered by increasing |*r*| (strongest correlations on top). In (C), molecular formulas with AI_mod_ > 0.5 are outlined in red, demonstrating that many molecular formulas that are not classified as aromatics by MS (red polygon) show strong positive correlations with aromatic structural motifs, as defined by NMR.

The observed correlations in PCoA and in van Krevelen space suggest that NMR signals traditionally assigned to carbohydrate-like structures may instead reflect lignin-derived structures, which are major components of terrigenous DOM. Such compounds have aromatic and oxygen-rich aliphatic motifs and are decorated with methoxy groups but they typically lack ethoxy-, 1-methylalkoxy, and longer-chain alkoxy (alkyl > ethyl) groups as well as anomeric protons, which would exhibit cross-peaks in sections f, g, and j, respectively. Additionally, HIX correlated with both protons in the α-position to single-bonded oxygen and aromatic protons (Figure 4A,B), suggesting their prevalence in terrigenous samples, consistent with the production of lignin from vascular land plants.

This interpretation is further supported by the correlation of NMR sections and MS-based compound classification. NMR-derived structural features generally correlated well with MS-based compound groups associated with similar functional groups. The vicinal diol section (h) was a notable exception, showing positive correlation with aromatic MS compound groups (Figure S16, SI) and AI_mod_ (Figure S17, SI). This further supports the finding that the vicinal diol section (h) largely contains signals from structural motifs that are part of aromatic compounds, such as lignin-type structures.

It is important to note that absolute abundances derived from the two techniques are not directly comparable because NMR and FT-ICR-MS are based on different physical principles. In particular, the relative abundance of carbohydrates inferred from NMR and MS were anticorrelated (correlation of NMR section j to MS group “saccharides” in Figure S16, SI). Since the anomeric proton region (j) is highly indicative of carbohydrates, this suggests that ESI-FT-ICR-MS is poorly suited for detecting this compound class in DOM, consistent with only 0.04–0.68% of intensity-weighted molecular formulas assigned as saccharides (Table 2). The discrepancy between abundances of carbohydrates as detected by NMR and MS reflects both differences in the analytical window of each method and low negative ESI ionization efficiencies.^12^ NMR detects structural features, and FT-ICR-MS detects masses (molecular formulas) of whole compounds. For example, saccharides may exist as glycosides such as quercetin 3-*O*-α-L-arabinopyranoside (C_20_H_18_O_11_, H/C = 0.9, O/C = 0.55, Figure S19, SI), which occurs in various terrestrial plants including *Rhizophora mangle*.^48^ This compound would be partly detected as a carbohydrate by ^1^H NMR (6 of 11 NMR-detectable protons are part of the saccharide) but would not be classified as a saccharide based on its molecular formula due to its relatively low H/C and O/C values.

Overall, these findings underscore the complementary strengths of NMR and FT-ICR-MS analyses for DOM characterization: FT-ICR-MS provides detailed molecular formula information for a wide range of compounds but poorly detects saccharides. In contrast, NMR reveals individual structural motifs, even when they represent only a small part of a larger compound, such as the glycone in glycosides.

### 3.6 Dissolved Black Sulfur from Fluvial Sources and Sulfurized Aliphatic Compounds from Mangrove-Derived DOM

The abundances of sulfur-containing molecular formulas from the six transect samples showed a strong salinity-dependent trend (Figure 6B). Most molecular formulas correlated with salinity (*p* < 0.1) clustered into three distinct sulfur-containing groups with specific H/C and O/C values (*cf*. S2.7, SI). The first group, defined by H/C < 0.6 and O/C < 0.3, was tentatively assigned to polycyclic aromatics containing sulfur (dissolved black sulfur), likely originating from incomplete combustion of organic matter.^12,19^ The second group, with H/C 0.6 to 1.0 and O/C < 0.3, and the third group, characterized by H/C > 1.0 and O/C > 0.4, were tentatively assigned to highly unsaturated and aliphatic sulfur-containing compounds, respectively.

**Figure 6:**
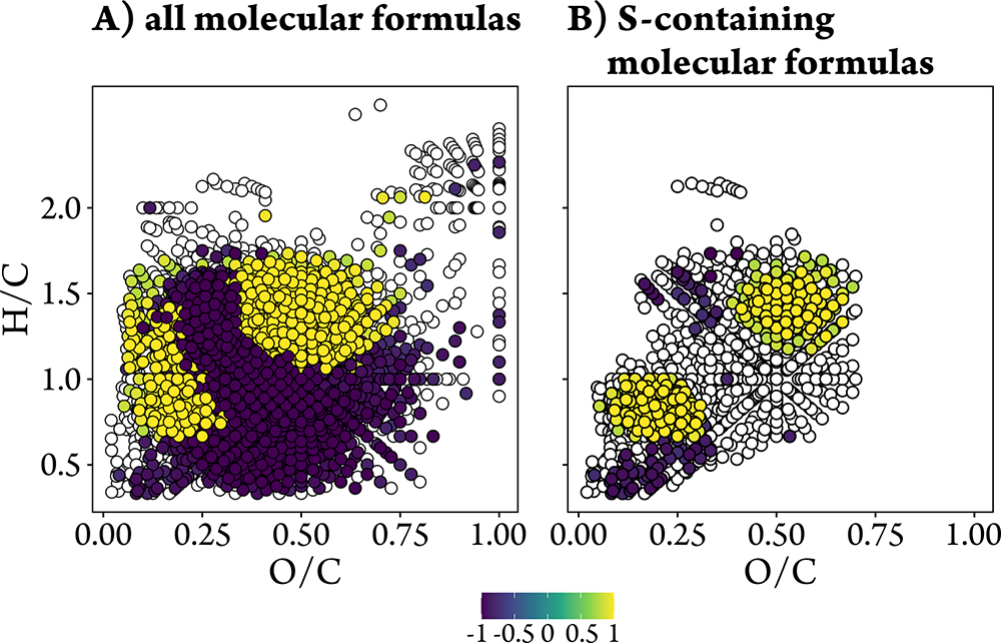
Van Krevelen diagrams displaying molecular formulas with positive (green to yellow) and negative (blue) correlations of molecular formula abundances with salinity across the six transect samples for (A) all molecular formulas and (B) sulfur-containing molecular formulas. All molecular formulas in the relevant subset are plotted as open white circles in the background, independent of the *p*-value. Molecular formulas with correlations considered significant (*p* < 0.1) are overlaid in the foreground, colored by the correlation coefficient (*r*) and layered by increasing |*r*| (strongest correlations on top). Three classes of sulfur-containing compounds were identified: (1) dissolved black sulfur (H/C < 0.6, O/C < 0.3), (2) highly unsaturated sulfur compounds (0.6 < H/C < 1.0, O/C < 0.3), 3) sulfurized aliphatic compounds (1.0 < H/C < 1.5, 0.4 < O/C < 0.7).

Our analysis of the relative molecular formula abundances of the respective groups provided information about their sources. The combined intensities of molecular formulas assigned as dissolved black sulfur decreased by almost 90% along the transect from river to coastal ocean (Table S7, SI), suggesting a predominant fluvial rather than mangrove-derived source.^49^ This interpretation is consistent with the long-standing practice of slash-and-burn agriculture in Brazil, which has led to the accumulation of soil charcoal that releases dissolved black carbon.^50^ In contrast, the abundance of highly unsaturated sulfur-containing compounds increased by ∼100% from riverine to coastal marine samples (Table S7, SI). We propose that these compounds may originate from degradation processes of dissolved black sulfur, resulting in the formation of sulfurized, highly unsaturated compounds with higher H/C but similar O/C values. Another distinct pattern was observed for aliphatic sulfur-containing compounds, which reached a relative maximum in the mangrove section of the transect (Table S7, SI). In the mangrove tidal creek, their relative abundance was almost three times higher than in any other sample. Notably, the intensity-weighted average sulfur content determined by FT-ICR-MS was approximately twice as high in the tidal creek samples (Table S2, SI) and was positively correlated with COSY section e, being indicative of *N*- and *S*-containing compounds (Figure S17, SI). This spatial pattern suggests that these compounds originate from abiotic sulfurization of dissolved and sedimentary organic matter,^51,52^ a process well-documented in sulfidic mangrove sediments.^13^ The observed increase in *S*-containing molecular formulas in the mangrove-fringed region, followed by their decline toward the coastal ocean, is consistent with our broader observation that structural diversity increased as molecular formula diversity diminished.

### 3.7 Characteristic Structural Features and Molecular Formulas in Marine and Terrestrial DOM

The statistical integration of NMR and MS data provided complementary insights into the DOM composition by combining molecular formula information (FT-ICR-MS) with structural motif information (NMR). Canonical correlation analysis (CCorA) revealed a strong correlation between the first canonical axes of the two data sets (Figure 7, *R* ^2^ = 0.97, permutational *p* = 0.04).

**Figure 7:**
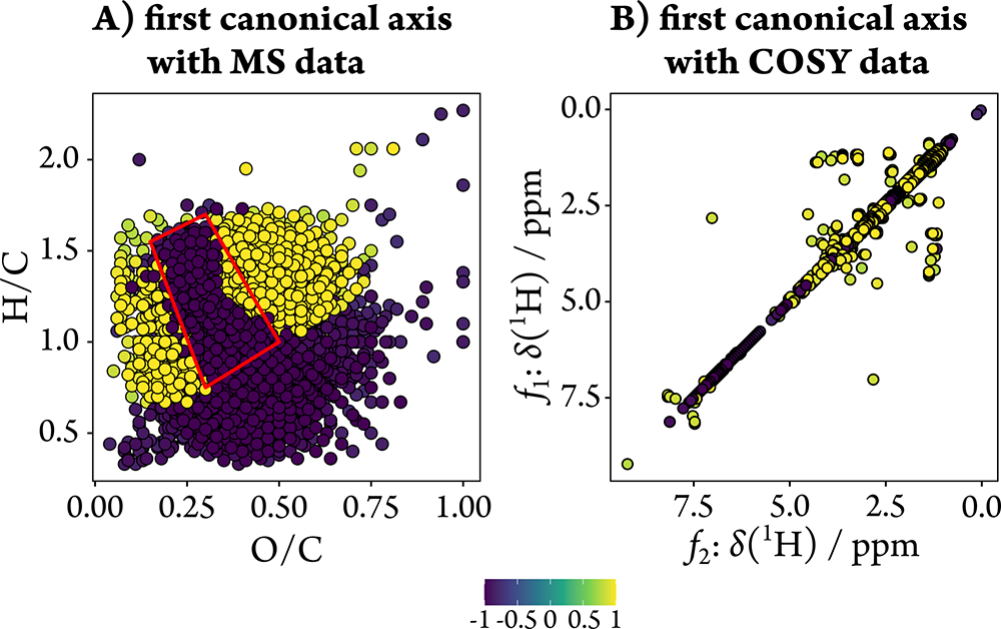
CCorA of the river-to-coastal ocean transect samples (*n* = 6) using the first two PCs of the PCoA based on MS and NMR data, respectively. Displayed are the color-coded correlations of the first canonical axis with the relative intensities of either (A) MS or (B) NMR data. Structural features colored in yellow (violet) in the simplified COSY NMR spectrum are associated with molecular formulas highlighted in yellow (violet) in the van Krevelen diagram and *vice versa*. Significance of canonical correlations was calculated as permutational probability associated with Pillai’s trace statistics with 719 permutations (*p* = 0.04).

Positively correlated molecular formulas, characterized by comparatively higher H/C values in the van Krevelen space, were primarily linked to heteroatom-substituted aliphatic moieties (yellow dots in Figure 7). In contrast, negatively correlating molecular formulas (violet dots, with comparatively lower H/C values) corresponded mainly to diagonal signals indicative of acetyl/aromatic methyl groups (2.1–2.5 ppm), methoxy groups/methyl esters (3.4–4.0 ppm), and aromatic/olefinic moieties (5.0–7.5 ppm). Notably, almost no off-diagonal signals were negatively correlated (violet dots).

Most signal intensity in the COSY spectra originates from diagonal peaks, representing the 1D ^1^H NMR spectrum (S2.4, SI). Interestingly, most negatively correlating signals typically linked to carbohydrates (3.4–4.0 ppm) corresponded to compounds lacking saccharide-like motifs, as indicated by minimal negatively correlating cross-peaks in this region. This is consistent with our PCoA and correlation analyses (*cf*. 3.5), suggesting that these signals primarily originate from nonsaccharide compounds. Likely candidate compounds contributing to negatively correlating signals include modified flavonoids or lignin degradation products (Figure S20, SI), which show few or no cross-peaks (Figure S21, SI). Acetylated lignin monomers are widespread in angiosperms,^53,54^ and flavonoids with methylated or acetylated aromatic groups occur in mangrove trees (e.g., *Rhizophora* and *Avicennia*).^48,55,56^ However, the prevalence of methoxy signals supports a predominance of lignin-related compounds, as methoxy groups are more characteristic of lignin than of tannin-derived structures.

We also observed a distinct cluster of negatively correlating molecular formulas (H/C: ∼0.7 to ∼1.6, O/C: ∼0.2 to ∼0.4) in the van Krevelen space (Figure 7A, red polygon). We propose that this pattern is a mangrove-related molecular signature, as a similar pattern has already been observed in a previous study.^57^ We further suggest that this pattern reflects the relative decrease in lignin- and tannin-rich terrigenous DOM, driven by the elevated release of sulfurized, highly unsaturated, and aliphatic compounds in mangrove-influenced waters. This is also evident from the correlation pattern of molecular formulas with salinity (Figure 6A), which closely mirrors the observed pattern, indicating that the transition from fluvial and mangrove to marine DOM was the primary driver of variability between data sets. Essentially the same molecular distribution can also be generated by correlating COSY section e (including *N*- and *S*-substituted aliphatic compounds) with the molecular formula intensities (not shown), again pointing to the contribution of sulfurized aliphatic compounds from mangrove sources. Consequently, negatively correlated, more unsaturated formulas are indicative of terrestrial DOM, while positively correlated, generally more saturated formulas represent marine DOM, a pattern commonly observed for river-to-ocean transects.^34^

The overall positive correlation of COSY cross-peaks with MS-derived molecular formulas may partly reflect the low number of cross-peaks in the river sample (*cf*. Figure S3, SI), thus driving the multivariate statistical analysis. As discussed in Section 3.3, missing cross-peaks could arise if many signals fell below the NMR detection limit due to structural diversity. However, comparison of diagonal and cross-peak intensity distributions across all COSY spectra (S2.4, SI) showed no evidence for disproportionately weaker cross-peaks in the river sample. Since sample concentrations and total signal intensities were comparable, this argues against a dynamic range artifact. Instead, the river sample likely contained predominantly compounds with similar chemical motifs, consistent with lignin-derived material, which would produce fewer cross-peaks. An alternative explanation is that only a few abundant compounds in river DOM generated detectable cross-peaks, while the remainder were structurally diverse but too weak to register. Yet this scenario would imply an unusually uneven structural distribution, contradicted by the 1D NMR spectrum, which shows the broad, diffuse features typical of highly complex mixtures rather than the sharp peaks expected for samples dominated by a few species.

### 3.8 Overarching Insights into DOM Processing in Estuaries

Our results support the idea of higher structural diversity in marine compared to terrestrial DOM, showing that DOM structural diversity increases with salinity in the river-to-coastal ocean transect. Notably, the relative increase in structural diversity was more than threefold larger than the decline in molecular formula diversity (*cf*. Figure 3), highlighting the opposing nature of structural and compositional changes. Likely drivers for increasing structural diversity are oxidation processes of oxygen-containing structural motifs, such as the conversion of hydroxy groups to ketones, aldehydes, or carboxyl groups. Additionally, we observed unique molecular signatures of sulfur-containing compounds along the transect. Dissolved black sulfur, which is presumably derived from biomass charring during wildfires, decreased along the transect, likely due to degradation into compounds with higher H/C but similar O/C values. In contrast, sulfidic mangrove porewaters promoted the abiotic sulfurization of organic matter, particularly the formation of aliphatic sulfur-containing compounds. Meanwhile, aromatic moieties decreased, while aliphatic moieties increased from the river to the ocean, reflecting the preferential photooxidation and removal of terrestrial aromatic motifs as well as the production of more aliphatic structures by marine primary producers. An increased aliphatic contribution renders compounds more nonpolar and devoid of π-systems, which reduces their tendency to adsorb onto mineral surfaces or colloids and form complexes with metals in water. As a result, DOM probably becomes more mobile, less susceptible to photochemical oxidation, and more persistent in the water column. These overarching patterns and their implications for microbial accessibility, transport, and carbon cycling are summarized in the conceptual model (Figure 8).

**Figure 8:**
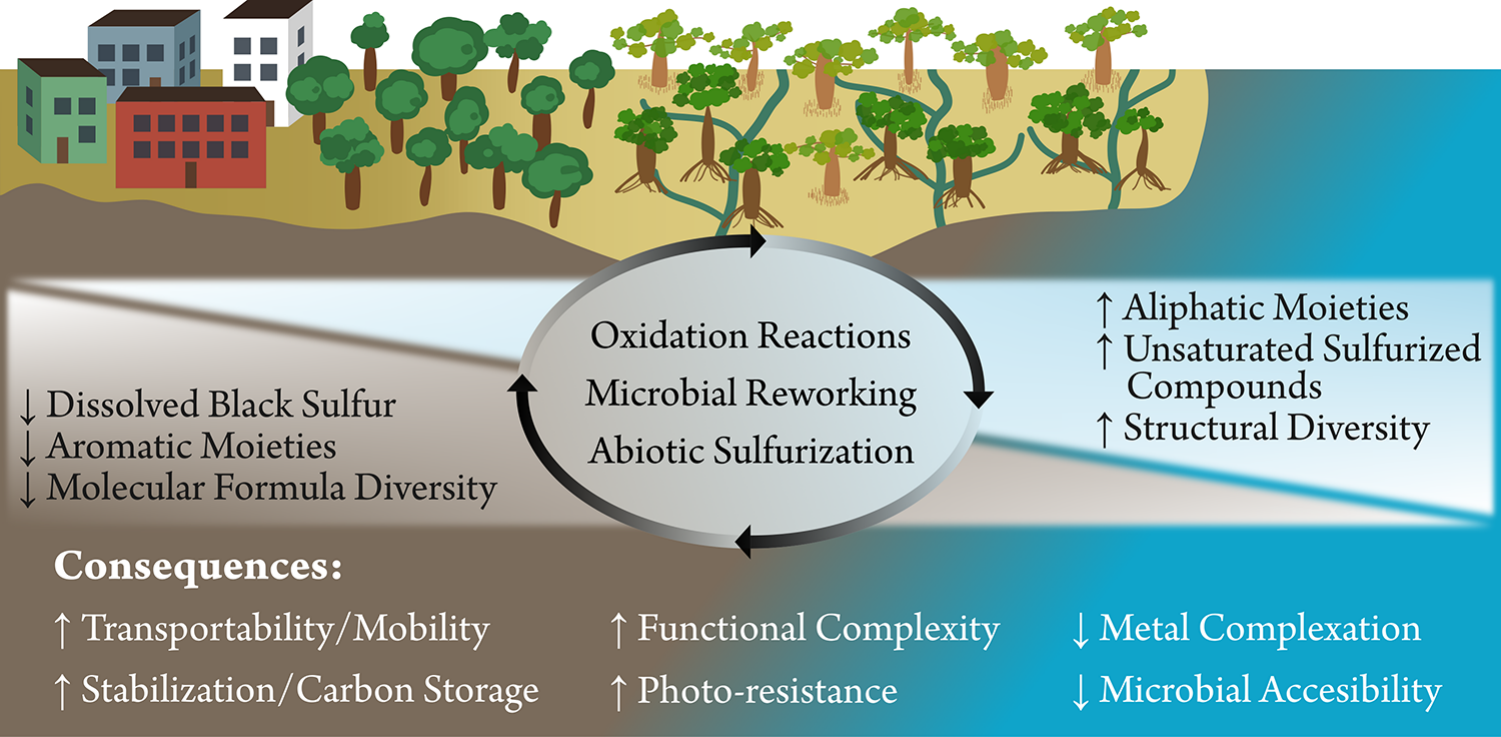
Conceptual model of DOM processing along a mangrove-fringed river-to-coastal ocean transect and its biogeochemical implications, emphasizing how nonspecific modification pathways and the buildup of structural diversity translate into large-scale impacts on the cycling and fate of carbon in aquatic systems. The top panel illustrates changes in DOM molecular features and the dominant processes driving these changes. The bottom panel links these molecular alterations to potential ecological and carbon cycle consequences.

An increase in oxygen content and, to some extent, a reduction in unsaturation within a given compound both lead to a larger number of potential structural isomers. When the processes driving these changes occur at least partly by nonspecific mechanisms, they inevitably result in greater structural diversity. The huge number of isomers per molecular formula for marine DOM^8,38^ and the stochastic behavior of certain chemical properties^58^ support this assumption. This suggests that nonspecific modification pathways, such as radical-driven or photochemical reactions, are likely key drivers of structural alteration in DOM. Abiotic sulfurization, particularly under sulfidic conditions in mangrove sediments, likely adds another layer of intrinsic stability by introducing functionalities that may reduce enzymatic accessibility for heterotrophic microbes. At the same time, such molecular modifications at the land-ocean interface likely foster emergent stability by increasing the structural diversity and functional complexity of marine DOM, probably reducing its biological accessibility and promoting its persistence against microbial turnover. Consequently, even when molecular formula diversity remains stable or decreases across different environments (as shown in this study), the underlying structural variability probably has significant ecological and climatic implications for the biogeochemical cycling of DOM.

## Supplementary Material



## Notes

Supplementary methods (S1): Sampling sites (S1.1); Selected environmental parameters and metadata (S1.2); Mass spectrometric analysis (S1.3); Selected fluorescence- and FT-ICR-MS-derived indices (S1.4); NMR spectroscopy (S1.5); NMR data processing in MATLAB (S1.6); Simulation of NMR spectra (S1.7); Integral sections representative for key structural features derived from 1D ^1^H NMR (S1.8); COSY sections (S1.9); Supplementary results (S2): 1D ^1^H NMR spectra (S2.1); Comparison of detected molecular formulas and detected bins with signal (S2.2), Removal of diagonal signals in ^1^H,^1^H COSY NMR spectra (S2.3); Comparison of diagonal and cross-peak intensities (S2.4); Spearman correlation of COSY section integrals, MS compound groups, and MS-derived molecular parameters (S2.5); Detection of saccharides by MS and NMR (S2.6); FT-ICR-MS-based relative abundance of sulfur-containing groups (S2.7); Prediction of NMR spectra resembling characteristic COSY NMR signals detected by CCorA (S2.8); Supplementary References (S3) (PDF)
