# Supplementary material for: Dissolved Organic Matter in the Coastal Ocean Is Structurally More Diverse Than in Terrestrial Systems, as Shown in an Amazonian Mangrove Estuary
Source: Environ Sci Technol. 2026 Feb 16;60(8):6264–75. doi: 10.1021/acs.est.5c10721 (PMC12961764; doi:10.1021/acs.est.5c10721)
Supplement: Supplementary file 1 [file es5c10721_si_001.pdf]

Supporting Information

Dissolved Organic Matter in the Coastal Ocean is Structurally More Diverse than in Terrestrial Systems, as Shown in an Amazonian Mangrove Estuary

Nico Mitschke[1,*](0000-0002-1043-7199), Thorsten Dittmar[1,2](0000-0002-3462-0107), Michael Seidel[1,*](0000-0003-0934-1939)

[1]Institute for Chemistry and Biology of the Marine Environment (ICBM), School of Mathematics and Science, Carl von Ossietzky Universität Oldenburg, Ammerländer Heerstraße 114–118, 26129 Oldenburg, Germany.

[2]Helmholtz Institute for Functional Marine Biodiversity (HIFMB) at the Carl von Ossietzky Universität Oldenburg, 26129 Oldenburg, Germany.

Email: nico.mitschke@uni-oldenburg.de

m.seidel@uni-oldenburg.de

*Authors to whom correspondence should be addressed

Table of contents

S1 Supplementary methods ... 3

S1.1 Sampling sites ... 3

S1.2 Selected environmental parameters and metadata ... 4

S1.3 Mass spectrometric analysis ... 5

S1.3.1 Intensity-weighted molecular parameters determined by FT-ICR-MS ... 6

S1.4 Selected fluorescence- and FT-ICR-MS-derived indices ... 6

S1.5 NMR spectroscopy ... 7

S1.6 NMR data processing in MATLAB ... 8

S1.7 Simulation of NMR spectra ... 12

S1.8 Integral sections representative for key structural features derived from 1D ^{1}H NMR ... 12

S1.9 COSY sections ... 13

S2 Supplementary results ... 17

S2.1 1D ^{1}H NMR spectra ... 17

S2.2 Comparison of detected molecular formulas and detected bins with signal ... 21

S2.3 Removal of diagonal signals in ^{1}H,^{1}H COSY NMR spectra ... 22

S2.4 Comparison of diagonal and cross-peak intensities ... 25

S2.5 Spearman correlation of COSY section integrals, MS compound groups, and MS derived molecular parameters ... 26

S2.6 Detection of saccharides by MS and NMR ... 29

S2.7 FT-ICR-MS-based relative abundance of sulfur-containing groups ... 30

S2.8 Prediction of NMR spectra resembling characteristic COSY NMR signals detected by CCorA ... 31

S3 Supplementary References ... 33

S1 Supplementary methods

S1.1 Sampling sites

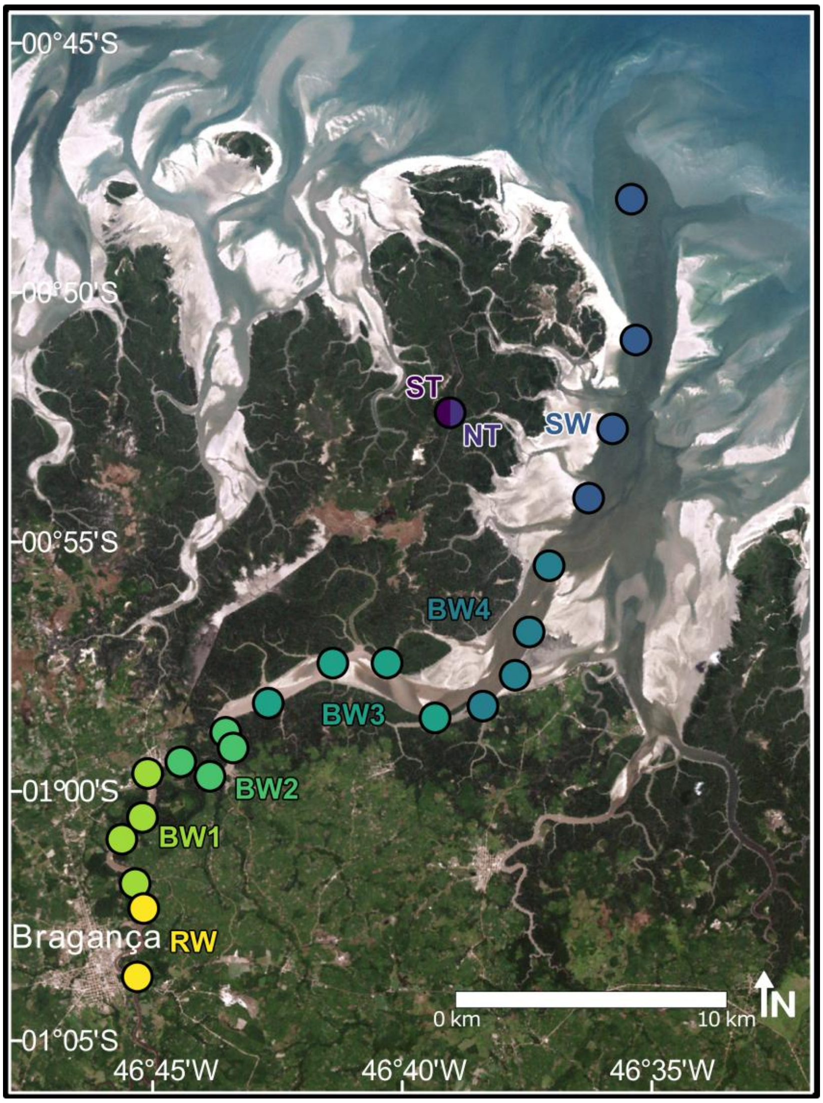

Figure S1 Sampling sites along the river-to-coastal ocean transect of the Caeté River in North Brazil (Pará State) and sampling site of the spring (ST, dark purple dot) and neap (NT, light purple dot) tide samples taken at low tide at the Furo do Meio creek. Samples taken at different stations along the transect were combined according to the different colors to six different samples that are designated as river water (RW, yellow dots), brackish water 1–4 (BW1–BW4, light green to turquoise dots) and seawater (SW, blue dots). Map modified after Knoke et al.[1]

S1.2 Selected environmental parameters and metadata

Table S1 Selected environmental parameters and metadata for the pooled samples, calculated as averages from the individual samples from Knoke et al.[1]

Sample	Salinity	$\delta^{13}C$ SPE-DOM [‰]	FDOM [rfu*]	DOC [µM]	TDN [µM]	DOC extraction efficiency [%]
Seawater (**SW**)	36.1	-23.8	16	110	8.7	61.8
Brackish water (**BW4**)	33.4	-25.6	50	188	14.6	62.3
Brackish water (**BW3**)	27.0	-26.4	83	242	23.3	65.8
Brackish water (**BW2**)	15.0	-27.5	94	245	30.8	68.0
Brackish water (**BW1**)	4.8	-28.5	76	203	26.9	71.7
River water (**RW**)	0.4	-29.1	66	187	17.9	70.4
Neap tide (**NT**)	35.1	-26.8	236	653	31.6	63.0
Spring tide (**ST**)	36.8	-26.6	134	512	41.4	65.9

*rfu: relative fluorescence units

S1.3 Mass spectrometric analysis

Analytical conditions and molecular formula attribution with ICBM-OCEAN are described in Knoke *et al.*[1] Briefly, methanol extracts were diluted to approx. 5 mg C L^{-1} with methanol/ultrapure water to a final ratio of 1:1 (methanol/water [*v*/*v*]) and directly infused into the ESI system (negative mode). The scan range covered 91 to 2000 Da and 200 scans were accumulated per sample. A mass accuracy of less than 0.1 ppm was achieved by internally calibrating the spectra with known molecular formulas covering the whole mass range.

In addition to the common compound group classifications implemented in ICBM-OCEAN,[2] molecular formulas were also classified as saccharides. Common criteria for the definition of saccharides by MS are, e.g., O/C ≥ 0.8, H/C ≥ 1.65, H/C < 2.7 and N = 0,[3] or O/C ≥ 0.7, O/C ≤ 1.1, H/C ≥ 1.5, H/C ≤ 2.4, N = 0 and P = 0[4]. The major drawback when using the O/C values or the presence of N or P as criteria is that many functionalized sugars do not meet, e.g., the conditions of O/C ≥ 0.8 or N = 0. Examples include amino sugars (e.g., glucosamine [$C_6H_{13}NO_5$, O/C = 0.83, H/C = 2.17, N = 1]; N-acetylglucosamine [$C_8H_{15}NO_6$, O/C = 0.75, H/C = 1.88, N = 1]), and *O*-methyl sugars (e.g., 3-*O*-methylrhamnose [$C_7H_{14}O_5$, O/C = 0.71, H/C = 2.00]). Thus, in this study, O/C ≥ 0.7, H/C ≥ 1.7 and H/C < 2.2 and no restriction regarding N, S and P were used as criteria.

For each detected molecular formula, the contents of carbon (C), hydrogen (H), oxygen (O), nitrogen (N), sulfur (S), phosphorus (P), the ratios of oxygen-to-carbon (O/C) and hydrogen-to-carbon (H/C), the aromaticity index (AI), the modified AI (AI_{mod})[5,6] and the double bond equivalents (DBE) were calculated. FT-ICR-MS data for the pooled samples were obtained as averages of samples used for pooling and deleting molecular formulas with intensities less than 1.5% of maximum intensity for each sample. Intensities of mass peaks with assigned molecular formulas were normalized to the sum of total signal intensities for each sample and multiplied by 10,000. Through this restriction, the number of attributed molecular formulas per sample was between 3778 to 6896. Finally, a matrix with the samples as rows and the intensity found for each molecular formula as columns was generated for further statistical analysis.

S1.3.1 Intensity-weighted molecular parameters determined by FT-ICR-MS

Table S2 Selected intensity-weighted molecular parameters as determined by FT-ICR-MS analysis.

Sample	*m*/*z*	C	H	O	N	S	P	H/C	O/C	AI_{mod}
Seawater (**SW**)	372	18.8	23.5	7.4	0.30	0.06	0.004	1.24	0.39	0.29
Brackish water (**BW4**)	362	18.5	22.3	7.1	0.25	0.08	0.004	1.19	0.39	0.32
Brackish water (**BW3**)	359	18.4	21.8	7.0	0.22	0.08	0.003	1.17	0.38	0.34
Brackish water (**BW2**)	365	18.9	22.1	7.0	0.19	0.08	0.005	1.16	0.38	0.35
Brackish water (**BW1**)	374	19.3	22.2	7.3	0.16	0.06	0.005	1.14	0.39	0.36
River water (**RW**)	388	20.0	22.8	7.6	0.16	0.06	0.017	1.13	0.39	0.37
Neap tide (**NT**)	391	19.4	22.2	8.0	0.20	0.17	0.003	1.14	0.41	0.34
Spring tide (**ST**)	410	19.9	22.8	8.8	0.20	0.17	0.001	1.14	0.45	0.32

S1.4 Selected fluorescence- and FT-ICR-MS-derived indices

Table S3 Selected indices for the pooled samples, calculated as averages from the individual samples from Knoke et al.[1]

Sample	HIX*	BIX*	I_{SuP}⁺
Seawater (**SW**)	0.76	1.05	0.09
brackish water (**BW4**)	0.87	0.79	0.15
Brackish water (**BW3**)	0.90	0.69	0.19
Brackish water (**BW2**)	0.90	0.68	0.19
Brackish water (**BW1**)	0.89	0.70	0.17
River water (**RW**)	0.88	0.63	0.15
Neap tide (**NT**)	0.92	0.65	0.25
Spring tide (**ST**)	0.88	0.73	0.23

*Fluorescence-derived DOM parameters: HIX[7] (humification index), BIX[8] (biological index)
⁺FT-ICR-MS-derived parameters: I_{SuP}[1] (molecular formula proxy for sulfidic porewater input)

S1.5 NMR spectroscopy

NMR spectra (1D ^{1}H and 2D ^{1}H,^{1}H COSY) were acquired with a Bruker AVANCE NEO 800 MHz instrument (Bruker Biospin GmbH, Ettlingen, Germany), equipped with a 5 mm TCI CryoProbe with *Z*-gradient (Bruker Biospin GmbH). 1D ^{1}H NMR spectra were acquired using the "*zgps*" pulse sequence (Bruker Biospin GmbH) after 16 dummy scans with 256 scans, 125k time domain points, an acquisition time of 5.0 s and a relaxation delay of 3.0 s. ^{1}H,^{1}H COSY NMR spectra were acquired using a slightly modified version of the "*cosygpppqf*" pulse program (Bruker Biospin GmbH) after 16 dummy scans with 8 scans per increment and with 12930 (f_2) times 384 (f_1) datapoints. Acquisition times were set to 750 ms (f_2) and 45 ms (f_1), respectively and the relaxation delay to 2.25 s. 1D ^{1}H spectra were zero-filled to 262144 datapoints and multiplied with a window function (exponential multiplication with line broadening = 0.30) before applying the Fourier transform. The intensity scaling factor (NC_proc) was set to 11 for ^{1}H NMR spectra. Phase and baseline corrections were done manually. 2D ^{1}H,^{1}H COSY NMR spectra were resized to 4096 (f_2) times 2048 (f_1) datapoints by zero filling and multiplied with a sine-squared window function before applying Fourier transform. A lower resolution along f_2 with respect to the original datapoint size was selected since no information gain was expected due to the higher digital resolution and subsequent processing is faster with fewer datapoints. The intensity scaling factor (NC_proc) was set to 10 for ^{1}H,^{1}H COSY NMR spectra. All basic processing steps were performed with TopSpin (versions 4.1.4 and 4.3.0, Bruker BioSpin GmbH).

S1.6 NMR data processing in MATLAB

NMR data was stored in Bruker specific file formats and imported to MATLAB (version R2022b, The MathWorks, Inc.) using the Read_Bruker_1D and Read_Bruker_2D scripts as provided by the National Magnetic Resonance Facility at Madison (NMRFAM, Univ. of Wisconsin-Madison, Wisconsin, USA, http://pine.nmrfam.wisc.edu/download_scripts.html). Along with the data, also the following parameters were imported:

a) yCAR and xCAR: carrier frequency (ppm) in f_1 and f_2, respectively
b) ySW and xSW: sweep width (Hz) in f_1 and f_2, respectively
c) yOBS and xOBS: resonance frequency (MHz) of the observed nucleus in f_1 and f_2, respectively
d) Length: number of datapoints (only imported for 1D data).

Initially the axes boundaries were calculated from the above-mentioned parameters as follows:

$$f_{\mathrm{min}} = \text{carrier frequency} - \frac{1}{2} * \frac{\text{sweep width}}{\text{resonance frequency}}$$

$$f_{\mathrm{max}} = \text{carrier frequency} + \frac{1}{2} * \frac{\text{sweep width}}{\text{resonance frequency}}$$

Subsequently, boundaries for each datapoint were calculated as linearly spaced vectors from f_{min} to f_{max} with N_{data}+1 points under the assumption that the axes values are equally distributed from f_{min} to f_{max} with their centers lying the middle of a datapoint. Axes values (represented by the centers of each datapoint) were calculated as the average of two adjacent boundary values. Subsequently, 1D and 2D data were processed differently.

Further processing of 2D NMR data

Noise removal:

Noise was deleted from 2D NMR spectra using a variable signal-to-noise (SNR) cutoff (SNR_{cutoff}). The SNR_{cutoff} can be set to any value but was set to three by default. To delete noise from spectra, first the root-mean-square (*rms*) of noise (rms_{noise}) was calculated from an (almost) signal-free region in the upper left corner of the spectrum. This region can be adjusted arbitrarily but was set to $f_{1,\mathrm{min}}$ to 5 ppm and 5.5 ppm to $f_{2,\mathrm{max}}$ by default. After calculation of rms_{noise} the SNR of all datapoints was calculated according to:

$$\mathrm{SNR} = \left| \frac{I_{\mathrm{datapoint}}}{2 * rms_{\mathrm{noise}}} \right|$$

and the intensity of all datapoints where $SNR \leq SNR_{\mathrm{cutoff}}$ was set to zero. Remaining negative values were also set to zero, because the COSY spectra as acquired in the present study are not phase sensitive and thus negative values should only arise from instrument noise (Gaussian noise) or artefacts (e.g., t_1 noise).

Usually only a reduced subset (sub-region) of datapoints is used for further analysis. For binning, this becomes mandatory to ensure that the size (in f_1 and f_2) of the new data matrix is dividable by the chosen bin sizes. A common section of interest for proton derived NMR spectra such as ^{1}H,^{1}H COSY may be from 0 to 10 ppm. By default, implemented bin sizes (sw_{bin}) in the MATLAB script were chosen to be 1.00. 0.50, 0.25, 0.20, 0.10, 0.05, 0.025, 0.02 and 0.01 ppm. Other bin sizes can be used but must be divisors of the bandwidth of the chosen sub-region (here 10 ppm) that still lead to integer numbers. The step size for sub-region selection must at least match the used bin size. To ensure this, the step size of subregion-selection was set to the largest implemented bin size of 1.00 ppm.

After the bin size has been chosen, the number of bins required to cover the whole selected sub-region was calculated for each dimension according to:

$$N_{bins}=\frac{f_{max,subregion} - f_{min,subregion}}{sw_{bin}}$$

and the binning boundaries were defined as a linearly spaced vector from $f_{min,subregion}$ to $f_{max,subregion}$ with a step size of sw_{bin}. Usually, binning boundaries will not be the same values as the data point boundaries. One approach to encounter this is to partition the intensity of datapoints that are "divided" by the binning boundaries according to their distance to the binning boundaries and the boundaries of the actual data points. For this, all data points that fall within the binning boundaries (default from 0 to 10 ppm, see above) were extracted and a vector with these datapoints and the binning edges (e.g., 0, 0.05, 0.1, 0.15, …, 9.95, 10.0 for sw_{bin} = 0.05) with all values sorted either in ascending or descending order was constructed. New axes were constructed from these vectors essentially as described above for the raw data as the center of two neighboring boundaries. Next, the nearest datapoints for each of the new datapoints within the old (not binned) axes were searched. For obvious reasons, all datapoints expect from those of the binning boundaries will be identical to the former data points. Due to the binning edges, some axis values will be assigned the same data points. To avoid the assignment of a datapoint to two axis values, the intensity values must be transformed by assigning the intensity to the new datapoints according to their distance to the old axis (*cf.* Figure S2, for clearness, the visualization is done using a 1D example; 2D data was processed analogously).

After this transformation step, the data is suitable for binning, which is done by summing up all transformed data points within two binning edges (bold values in Figure S2). The axes of binned data were calculated as the average of two adjacent binning boundary values. Binned ^{1}H,^{1}H COSY spectra of all samples are shown as examples with a bin size of 0.05 ppm in Figure S3.

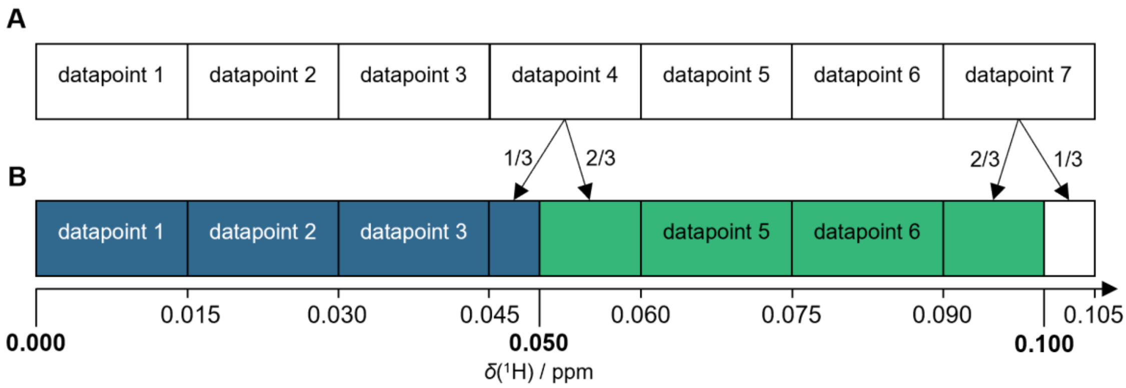

Figure S2 A) Original (not binned) datapoints. B) Transformed datapoints with binning boundaries (bold). The intensity of former data points 4 and 7 is assigned to two new data points and partitioned according to their proportion with respect to the old axes (here 1/3 and 2/3). Values are given in ppm.

Further processing of 1D NMR data

1D NMR data was processed analogously to 2D NMR data with the exception that no noise was deleted from the spectra and that solvent regions (4.70–5.05 ppm for H_2O and 3.20–3.45 ppm for MeOH) were removed from the spectra after data transformation. To ensure the solvent regions can be cut off precisely, the distinct ppm values of the solvent exclusion regions were added to the vector containing the data point and binning boundaries on which the data point transformation was conducted.

Statistical analysis of NMR spectra

Prior to statistical analysis, NMR data were sample-wise normalized by dividing the intensity of each data point (bin) by the total sum of intensities for that sample. Subsequent statistical analyses were performed on a data matrix structured with samples (observations) as rows and bins (variables) as columns. For 2D NMR data, the two axes were joint to represent the data point matrix as a vector (e.g., a spectrum represented by 200 x 200 data points after binning is now represented as a vector with 40,000 entries). For tracing back to specific chemical shift values, these were stored as the first two rows of the matrix. Finally, columns (corresponding to distinct bins) containing only intensity values of zero were removed. In the statistical approach applied in this study, the removal of zero-value intensities does not change the results but significantly cleans up the matrix. This is because all zero columns do not influence Bray-Curtis dissimilarity. Further statistical analyses were carried out as described in the main manuscript.

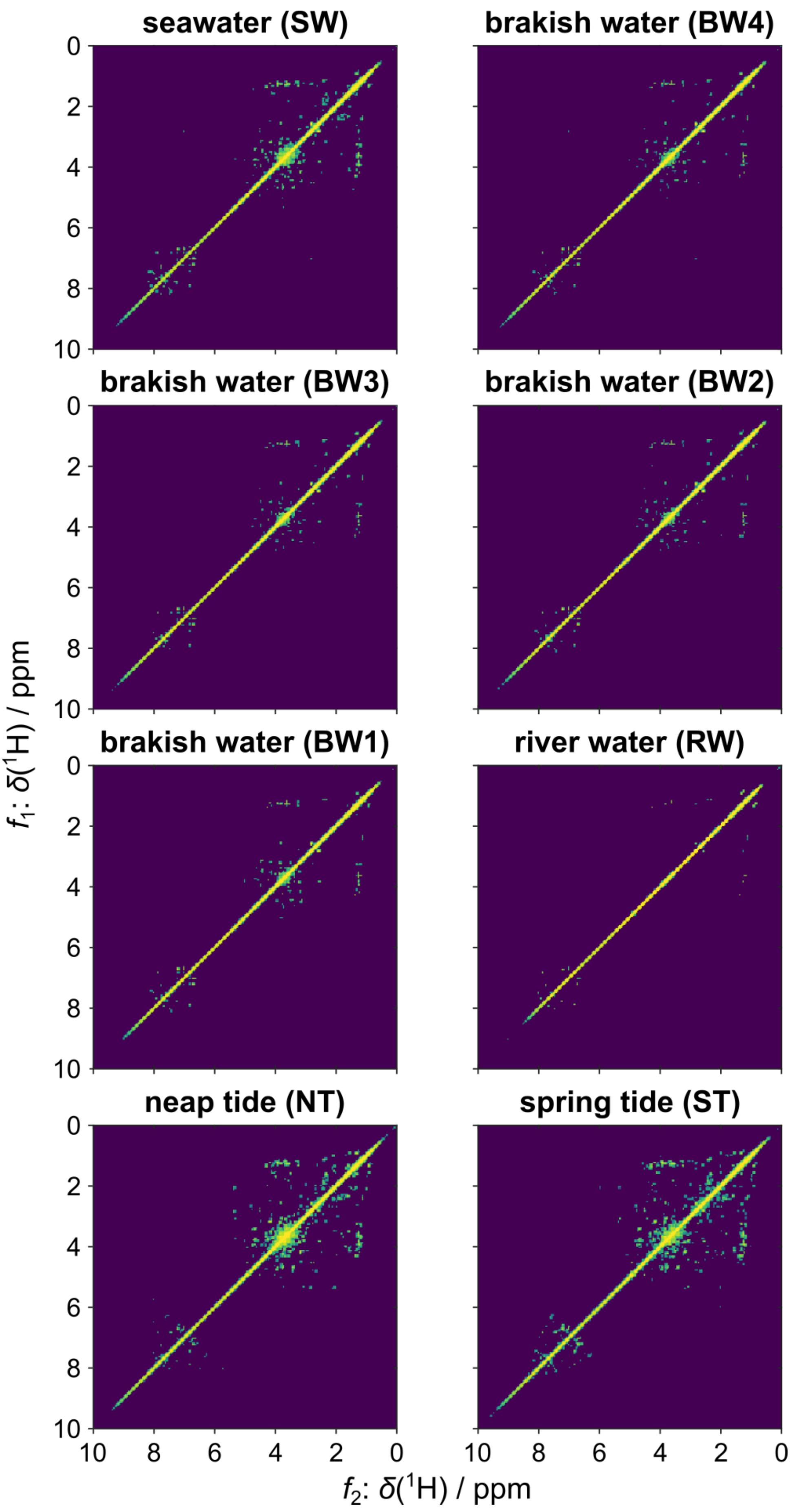

Figure S3 Binned $^1H,^1H$ COSY spectra of all samples using a bin size of 0.05 ppm.

S1.7 Simulation of NMR spectra

^{1}H,^{1}H COSY NMR spectra were predicted using the software ACD/NMR Workbook 2022.2.3 (Advanced Chemistry Development, Inc.). Prediction parameters were chosen to resemble the experimental parameters as closely as possible. The spectrometer frequency (for ^{1}H) was set to 800 MHz. The spectrum size was set to 2048 × 2048 points due to software constraints that only permit equal numbers of data points in both dimensions. The spectral range was set to -1 to 10 ppm for both dimensions and the line width was set to 1 Hz. All spectra were simulated for CD_3OD as the solvent using “corrected weighted average experimental” as the calculation algorithm.

S1.8 Integral sections representative of key structural features derived from 1D ^{1}H NMR

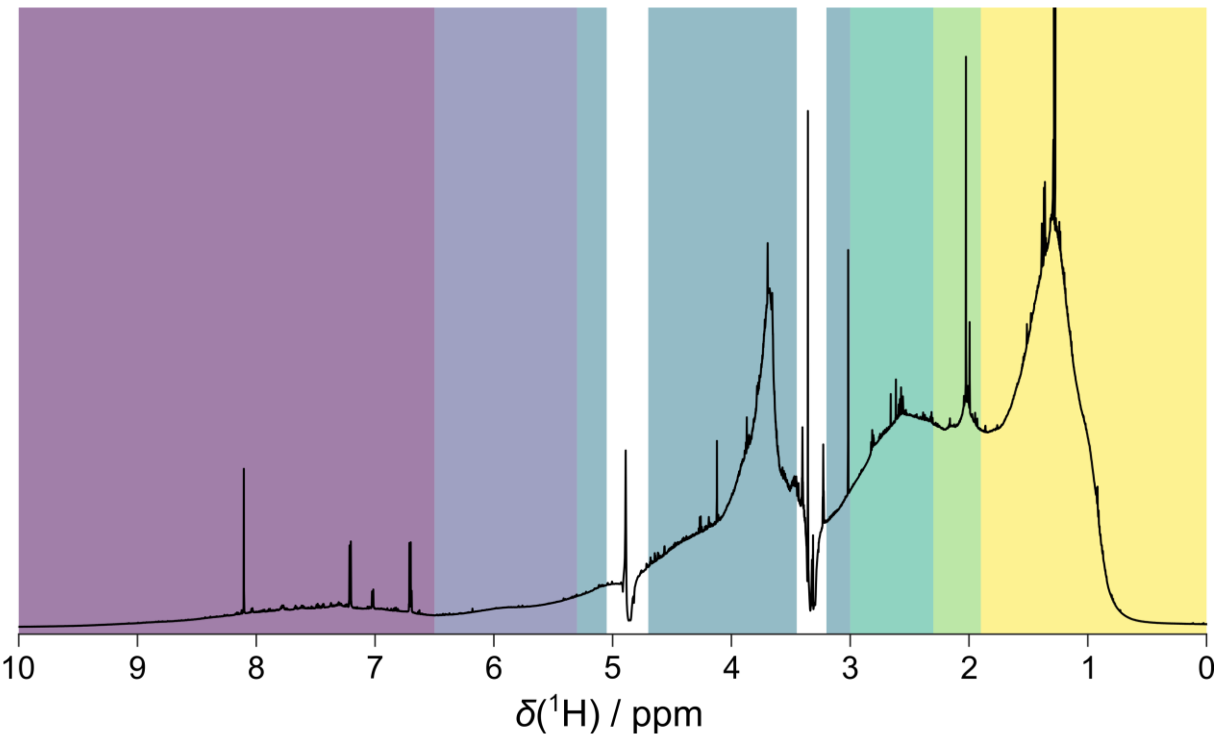

Figure S4 1D ^{1}H NMR spectrum (800 MHz, CD_3OD) of the seawater sample of the present study and division into six regions representing protons of distinct structural motifs. purple: aromatic protons (6.5–10.0 ppm), blue-violet: olefinic protons (5.3–6.5 ppm), blue: protons in the α-position to single-bonded oxygen (3.0–5.3 ppm), teal: protons in the α-position to carbonyls (2.3–3.0 ppm), green: acetyl protons (1.9–2.3 ppm), yellow: aliphatic protons (0.0–1.9 ppm). The uncolored sections from 4.7–5.05 ppm and 3.2–3.45 ppm represent solvent regions that were excluded from further analysis.

S1.9 COSY sections

We used the following assumptions and “rules” for establishing the COSY sections:

1) To create flexible and broadly applicable sections, it is advantageous to define more sections than may initially seem necessary. This allows for greater adaptability, as related sections can later be combined into broader parent categories. For example, all sections representing aromatic protons, such as pure aromatics, aromatics with electron-donating groups, etc., can be combined into a single category like “total aromatics”. There are several ways to combine sections. The most obvious way is purely additive. However, one might attribute equal importance to each combined section by rescaling of sections. This can be done by rescaling combined sections to a common scale such as zero to one, before summing their intensities. Since different sections usually also span varying spectral ranges, another normalization step with respect to the covered spectral ranges may be desired in certain cases. In this study, we used an additive approach and only applied a symmetry correction (i.e., section intensities mirrored across the diagonal were treated as redundant and counted only once).
2) At the same time, it is important to maintain clarity and define only as many sections as really needed. For example, no individual sections were established for aromatic protons of polycyclic hydrocarbons coupling to olefinic protons, pure aromatic protons coupling to olefinic protons, and protons of aromatic motifs substituted with electron-donating groups coupling to olefinic protons. Instead, these groups were combined into a single section representing all aromatic protons coupling to olefinic protons. This decision was primarily based on the fact that such structural motifs are relatively uncommon in DOM, making signals in these regions less likely to appear. Additionally, couplings between aromatic and non-aromatic protons must represent ^4J correlations, which are less commonly observed in COSY experiments compared to ^3J correlations.
3) In theory, drawing rigid boundaries between spectral sections is a simplification of the true NMR spectroscopic molecular properties. For instance, olefinic protons generally may resonate between 7.0 and 4.5 ppm depending on their substitution pattern. This range overlaps with aromatic protons, which typically appear between 10.0 to 6.5 ppm, as well with saccharides, whose anomeric protons may resonate downfield up to about 5.5 ppm. As a consequence, defining spectral sections is inherently a compromise to enable (semi-)quantitative analysis of complex NMR spectra. In the case of DOM, anomeric protons with chemical shifts > 5.0 ppm are expected to be more abundant than olefinic protons with chemical shifts < 5.3 ppm. Consequently, the olefinic region was defined from 6.5 to 5.3 ppm.

4) All types of exchangeable protons were excluded from sectioning because samples are usually measured in protic solvents such as methanol-d_4 or D_2O.
5) Common COSY spectra are not inherently quantitative. This is particularly true when the diagonal is included in the analysis because the majority of the signal intensity is concentrated along the diagonal, which is essentially the projection of the 1D ^{1}H NMR spectrum. For our samples, we found that > 97% of the signal intensity is stored along the diagonal (*cf*. Table S6). Therefore, particular attention must be paid when comparing absolute intensity values between different sections within the same sample (referred to here as intra-sample comparison).
6) Sections are named after the key structural features that are expected to be most abundant in DOM. However, it should be noted that other structural features may also contribute signals within the same section.

The sectioning scheme applied in the present study and related key structural features are summarized in Table S4.

Table S4 COSY sections as shown in Figure 2 of the main manuscript. Only selected examples of key structural motifs are included. CH_2 and CH groups are generally interchangeable. Residual groups indicate only the local substituent environment (e.g., "Alk" refers to the next ~two structural units). Structural motifs involving correlations with at least one exchangeable proton are not considered. Additional common motifs (e.g., allylic, halogenated, and other heteroatom-substituted fragments) are not shown explicitly to keep the table concise and because they are expected to be of minor importance in DOM.

#	δ_H (f_2) in ppm	δ_H (f_1) in ppm	Selected key structural motif	Example(s)
a	0.0–10.0<br>0.0–0.5	0.0–0.5<br>0.5–10.0	cyclopropyl motifs	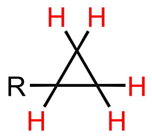
b	0.5–2.1<br>0.5–1.2	0.5–1.2<br>1.2–2.1	pure aliphatic methyl groups	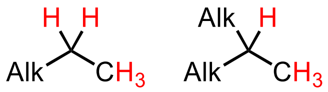
c	1.2–2.1	1.2–2.1	aliphatic methylene and methine connections	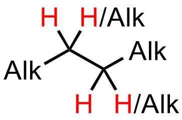
d	2.1–3.0<br>1.5–2.1	1.5–3.0<br>2.1–3.0	benzylic-, *N*/*S*-, and carbonyl-adjacent alkyl groups (alkyl > ethyl)	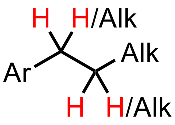 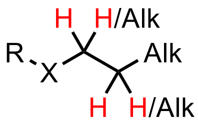<br>X = N, S, C(=O)
e	2.1–3.0<br>0.5–1.5	0.5–1.5<br>2.1–3.0	benzylic-, *N*/*S*-, and carbonyl-adjacent ethyl and 1-methylalkyl groups	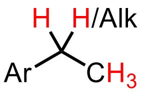 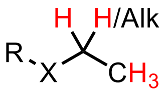<br>X = N, S, C(=O)
f	3.0–5.3<br>0.5–1.5	0.5–1.5<br>3.0–5.3	ethoxy- and 1-methylalkoxy groups	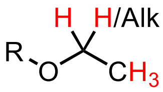
g	3.0–4.3<br>1.5–3.0	1.5–3.0<br>3.0–4.3	alkoxy groups (alkyl > ethyl)	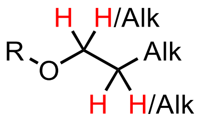
h	3.0–4.3	3.0–4.3	vicinal diols and their derivatives	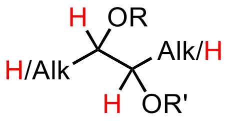 R, R' = H or organic residue
i	4.3–5.3<br>1.5–3.0	1.5–3.0<br>4.3–5.3	2-deoxy acetals	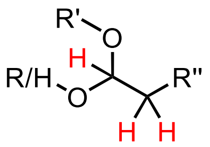 R'' ≠ O, N, S
j	4.3–5.3<br>3.0–4.3	3.0–4.3<br>4.3–5.3	anomeric protons of common saccharides	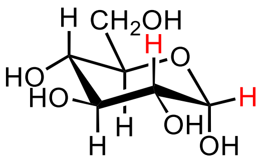

#	δ_H (f_2) in ppm	δ_H (f_1) in ppm	Selected key structural motif	Example(s)
k	4.3–5.3	4.3–5.3	acetal protons (self-correlation along diagonal)	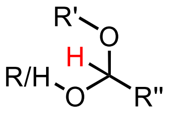
l	5.3–6.5	5.3–6.5	olefins	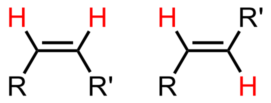
m	5.3–6.5<br>0.5–5.3	0.5–5.3<br>5.3–6.5	olefins attached to (modified) aliphatic motifs	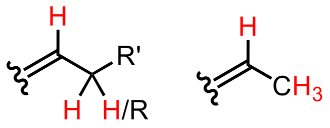
n	6.5–10.0<br>5.3–6.5	5.3–6.5<br>6.5–10.0	aromatics heavily substituted with EDG, EWG, and/or heteroatoms, occasionally olefins attached to aromatics (^4J correlation)	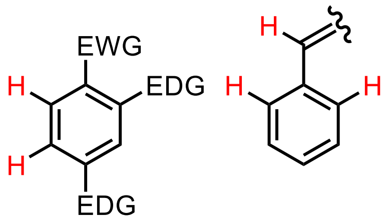
o	6.5–7.5<br>6.5–7.0	6.5–7.0<br>7.0–7.5	electron-rich aromatics	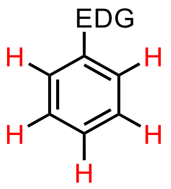
p	7.0–7.5	7.0–7.5	pure aromatics	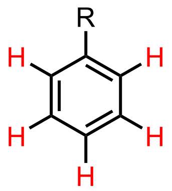 R ≠ EDG, EWG
q	7.5–10.0	7.5–10.0	PACs, electron-poor aromatics	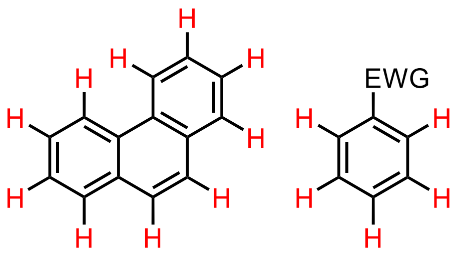
r	7.5–10.0<br>6.5–7.5	6.5–7.5<br>7.5–10.0	electron-rich PACs, aromatics substituted with EDG and EWG	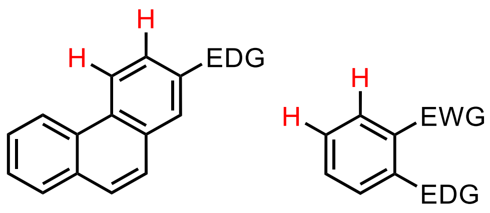
s	6.5–10.0<br>0.5–5.3	0.5–5.3<br>6.5–10.0	substituted aromatics (^4J correlations), aldehydes, and biomolecules (e.g., amides with sterically hindered and slowly exchanging protons)	

Alk: alkyl groups, Ar: aromatic substituents, R = generic substituent.
EDG: electron-donating group (e.g., $-NH_2$, -NHR, $-NR_2$, -OH, -OR).
EWG: electron-withdrawing groups (e.g., $-NO_2$, $-SO_3H$, $-SO_2R$, -CN, -CHO, $-CO_2H$, $-CO_2R$).
PACs: polycyclic aromatic compounds.

S2 Supplementary results

S2.1 1D ^{1}H NMR spectra

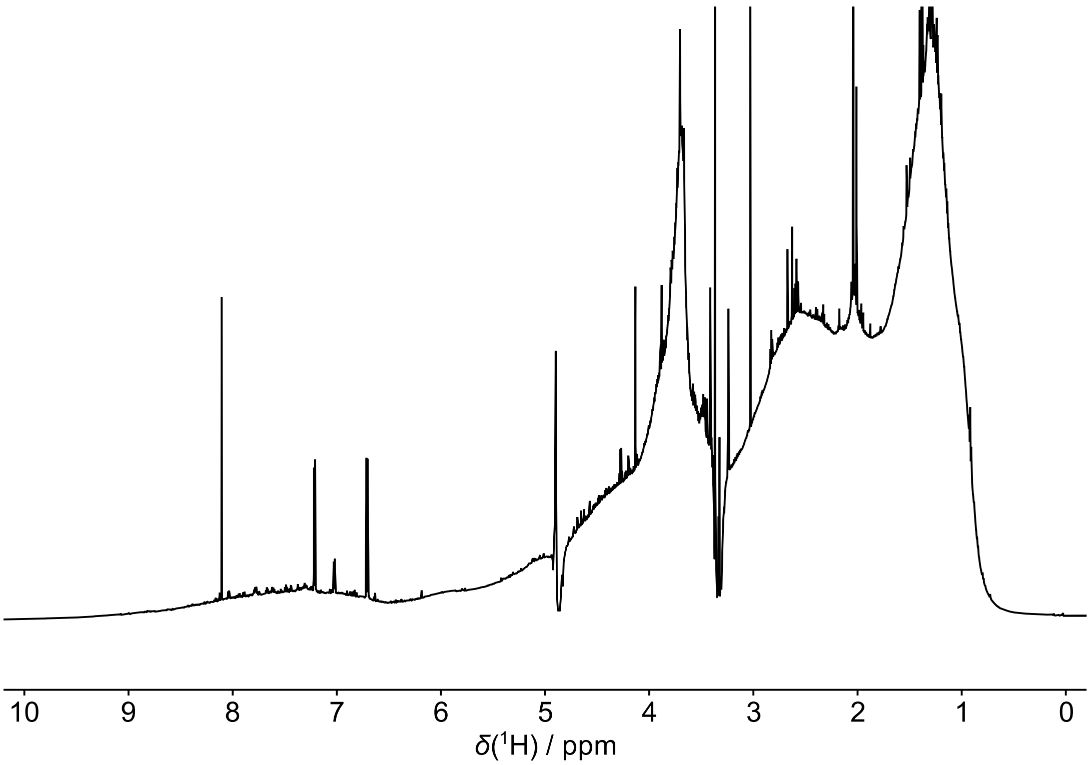

Figure S5 1D ^{1}H NMR spectrum (800 MHz, CD_3OD) of the seawater sample (SW).

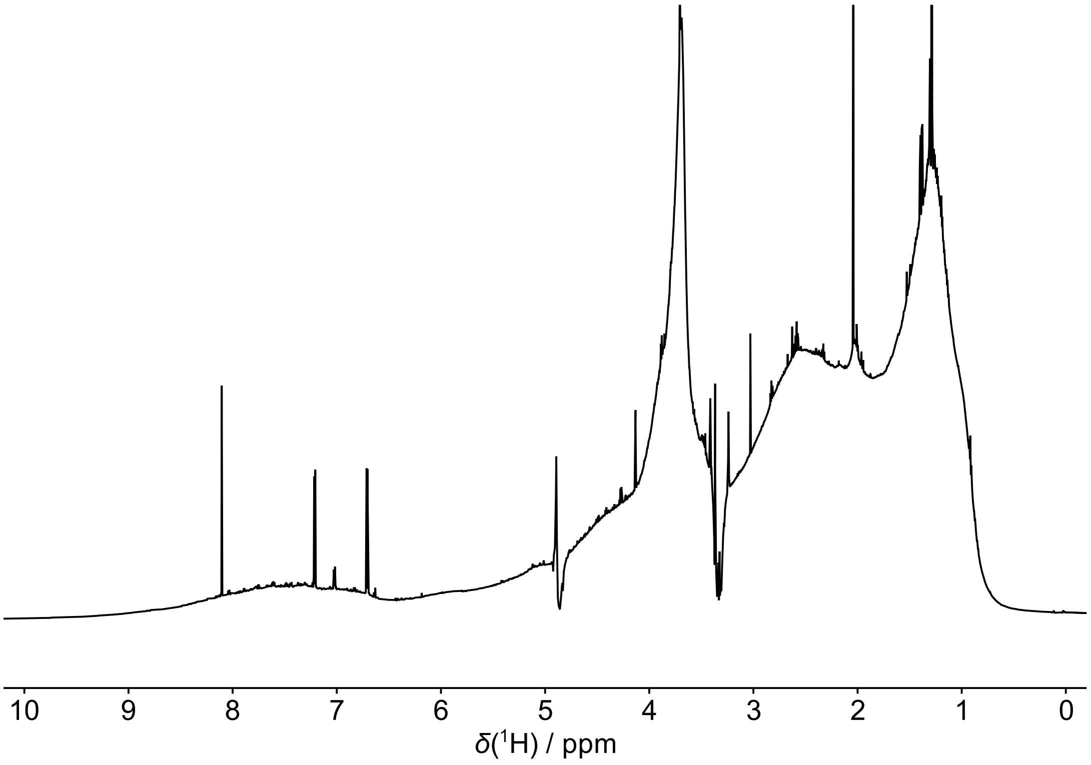

Figure S6 1D ^{1}H NMR spectrum (800 MHz, CD_3OD) of the fourth brackish water sample (BW4).

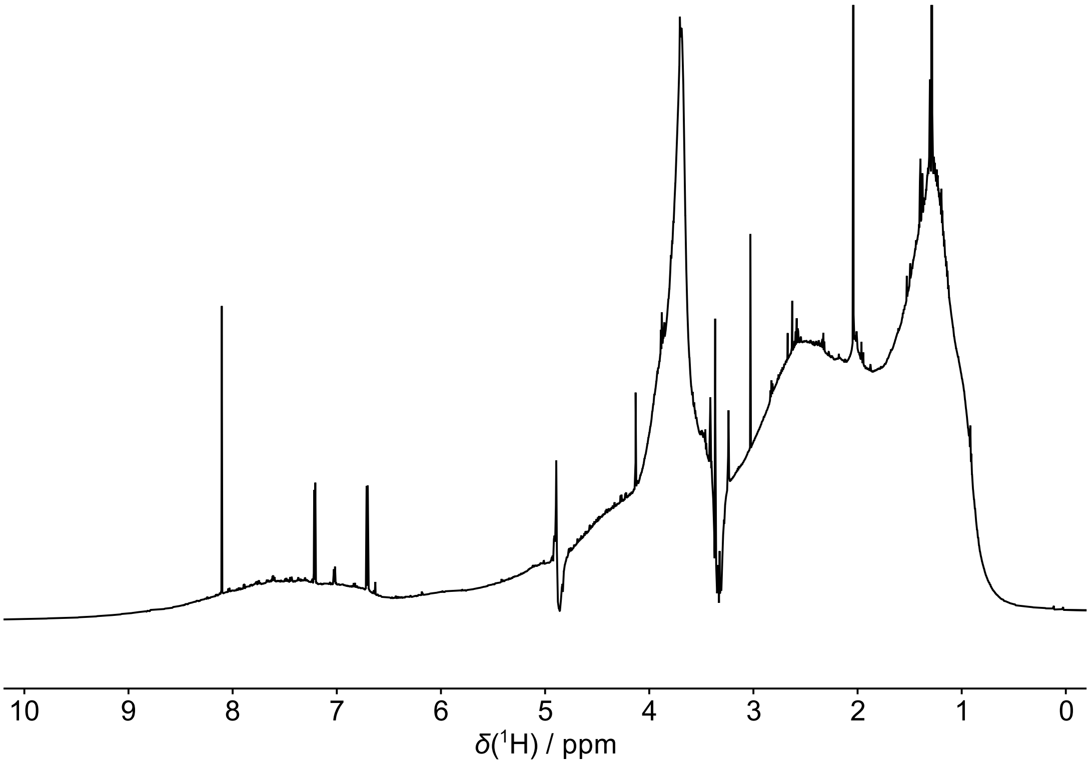

Figure S7 1D ^{1}H NMR spectrum (800 MHz, CD_3OD) of the third brackish water sample (BW3).

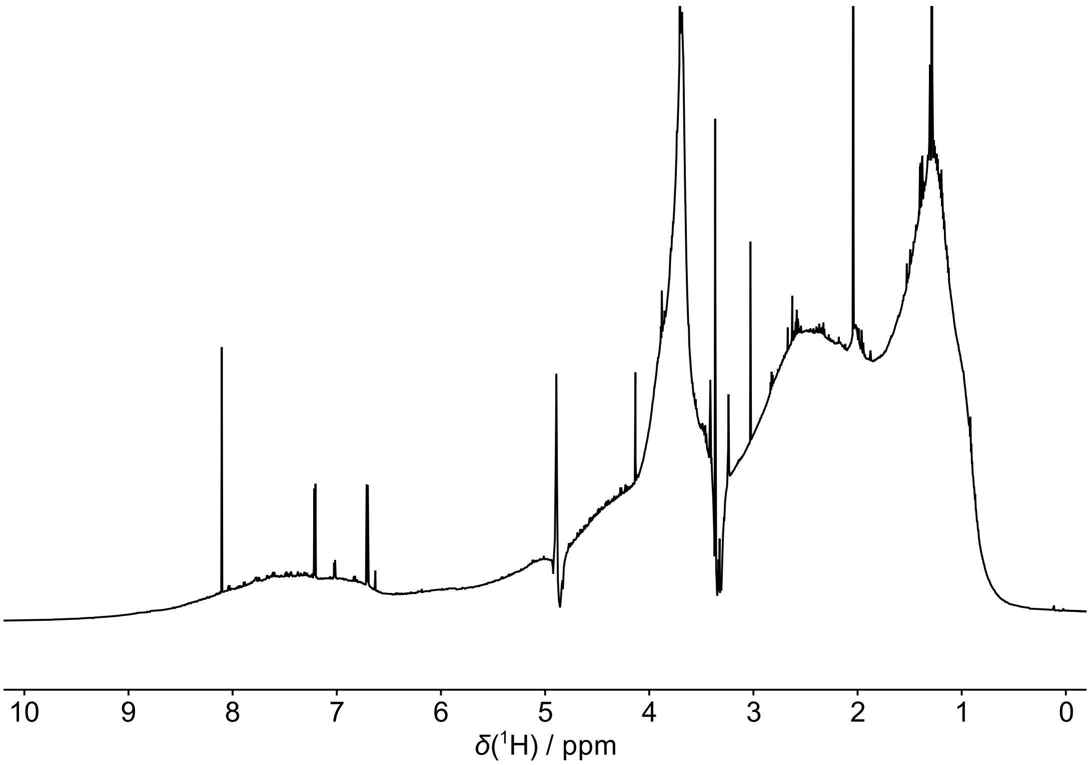

Figure S8 1D ^{1}H NMR spectrum (800 MHz, CD_3OD) of the second brackish water sample (BW2).

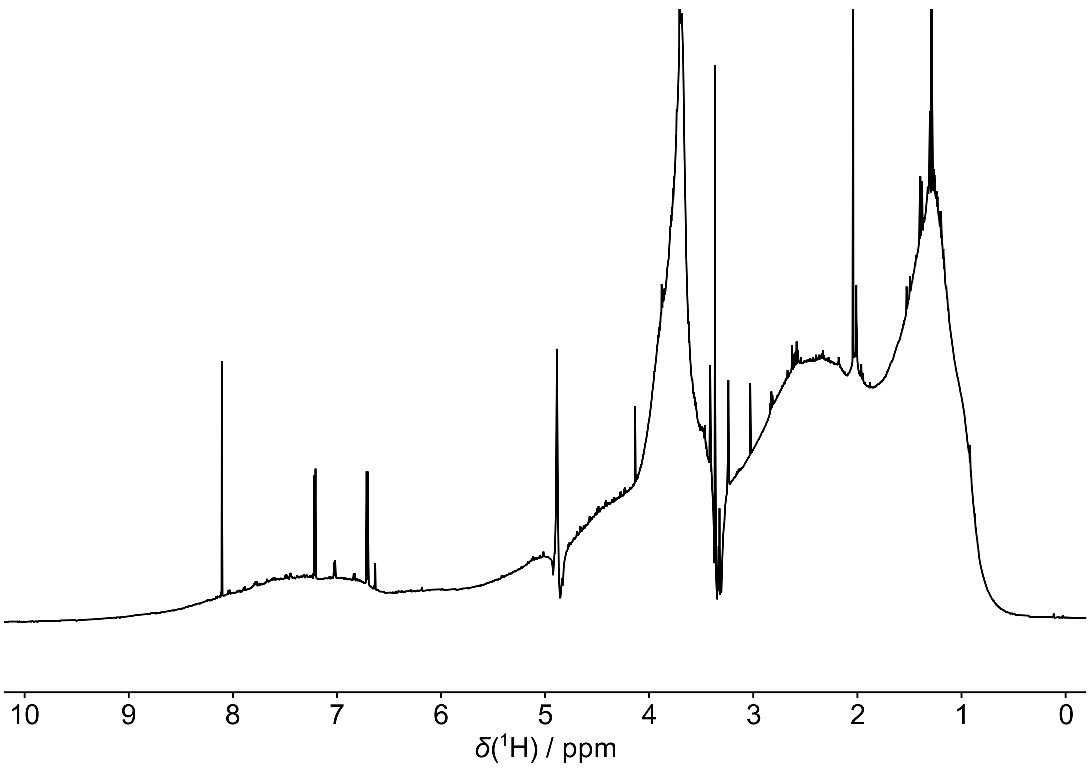

Figure S9 1D ^{1}H NMR spectrum (800 MHz, CD_3OD) of the first brackish water sample (BW1).

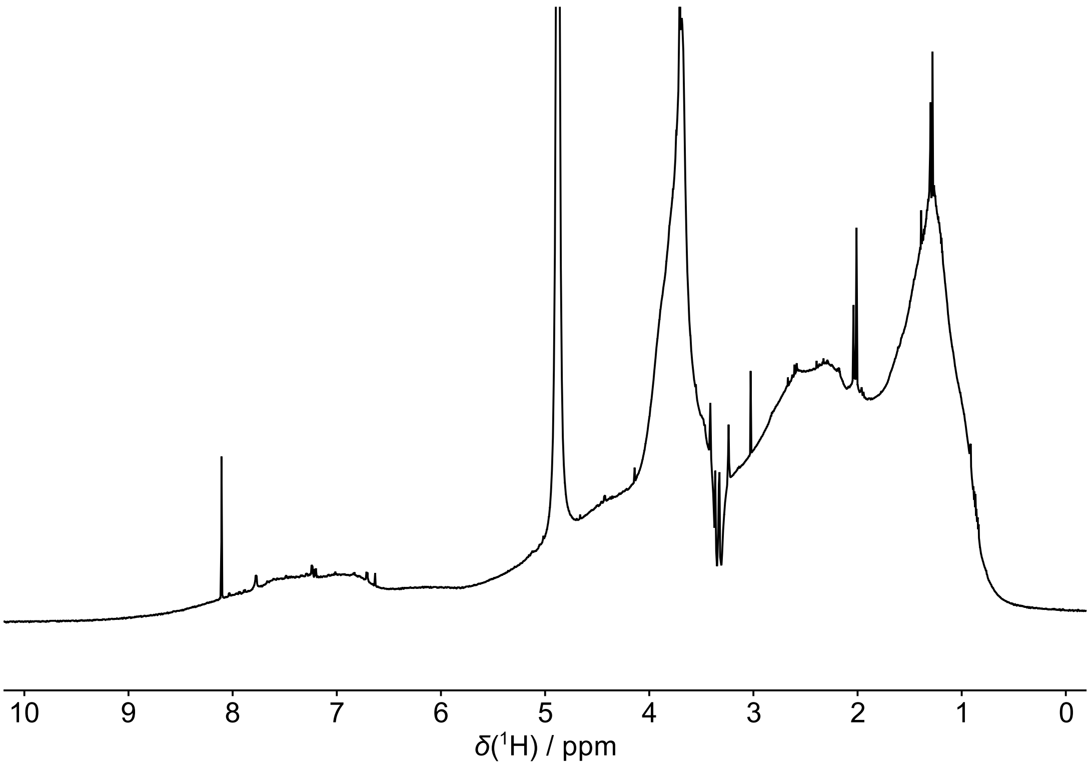

Figure S10 1D ^{1}H NMR spectrum (800 MHz, CD_3OD) of the river water sample (RW).

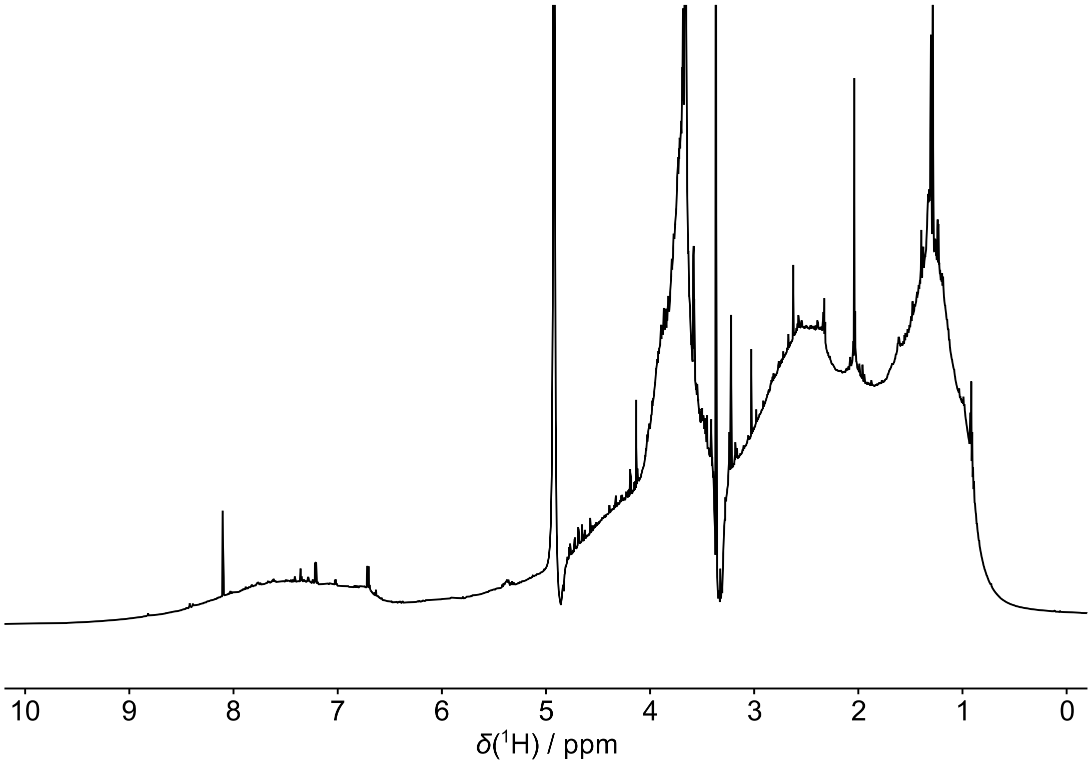

Figure S11 1D ^{1}H NMR spectrum (800 MHz, CD_3OD) of the neap tide sample (NT).

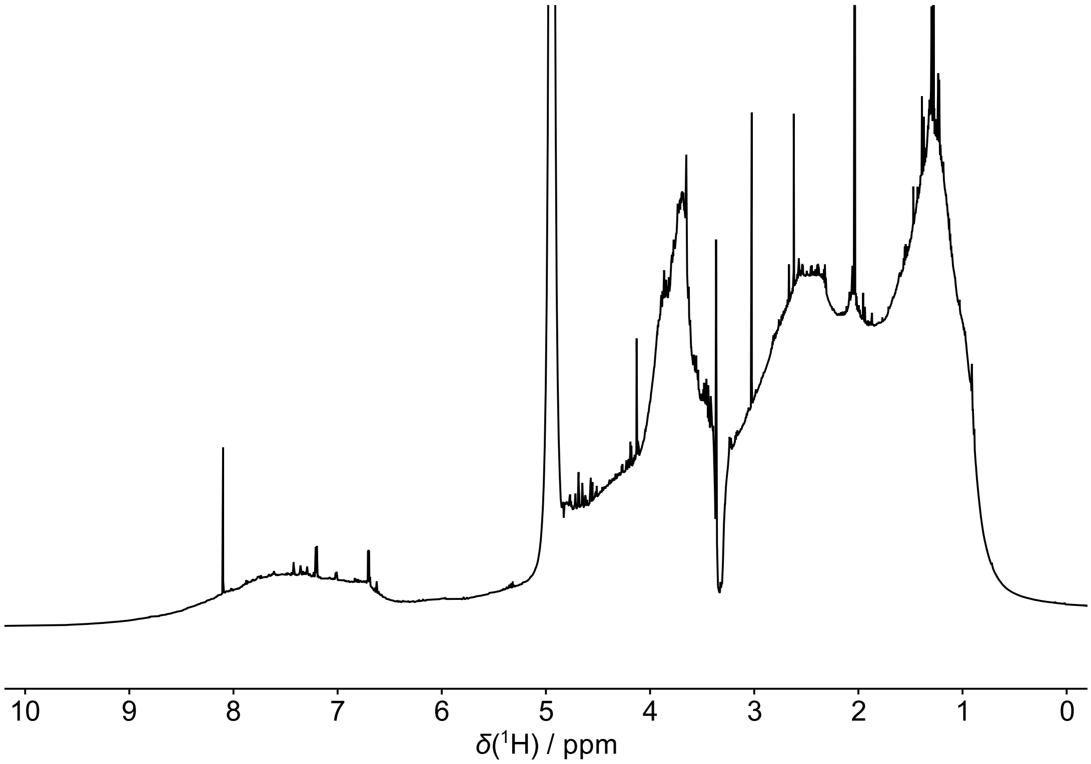

Figure S12 1D ^{1}H NMR spectrum (800 MHz, CD_3OD) of the spring tide sample (ST).

S2.2 Comparison of detected molecular formulas and detected bins with signal

Table S5 Number of detected COSY bins with signal and assigned molecular formulas for all samples. For comparison, “bins scaled” reports bin counts scaled by the seawater molecular-formulas-to-bins ratio.

Sample	Bins with signal	Molecular formulas	Bins scaled
Seawater (**SW**)	1302	3778	3778
Brackish water (**BW4**)	1026	4510	2977
Brackish water (**BW3**)	993	4562	2881
Brackish water (**BW2**)	999	5081	2899
Brackish water (**BW1**)	965	4948	2800
River water (**RW**)	624	5684	1811
Neap tide (**NT**)	1754	6896	5090
Spring tide (**ST**)	1925	6118	5586

S2.3 Removal of diagonal signals in ^{1}H,^{1}H COSY NMR spectra

For testing the influence of diagonal signals in the analysis of ^{1}H,^{1}H COSY NMR spectra, all diagonal signals were spaciously removed from the spectra. Signals were removed for a diagonal with a width of ~0.28 ppm, corresponding to a polygon spanned from 9.8/10.0 to 0.0/0.2 and 10.0/9.8 to 0.2/0.0 (f_1/f_2) ppm. This is illustrated in Figure S13 for the seawater sample. Bray-Curtis dissimilarities based on the binned COSY NMR data among samples are much larger for the COSY spectra without the diagonal signals (Figure S15).

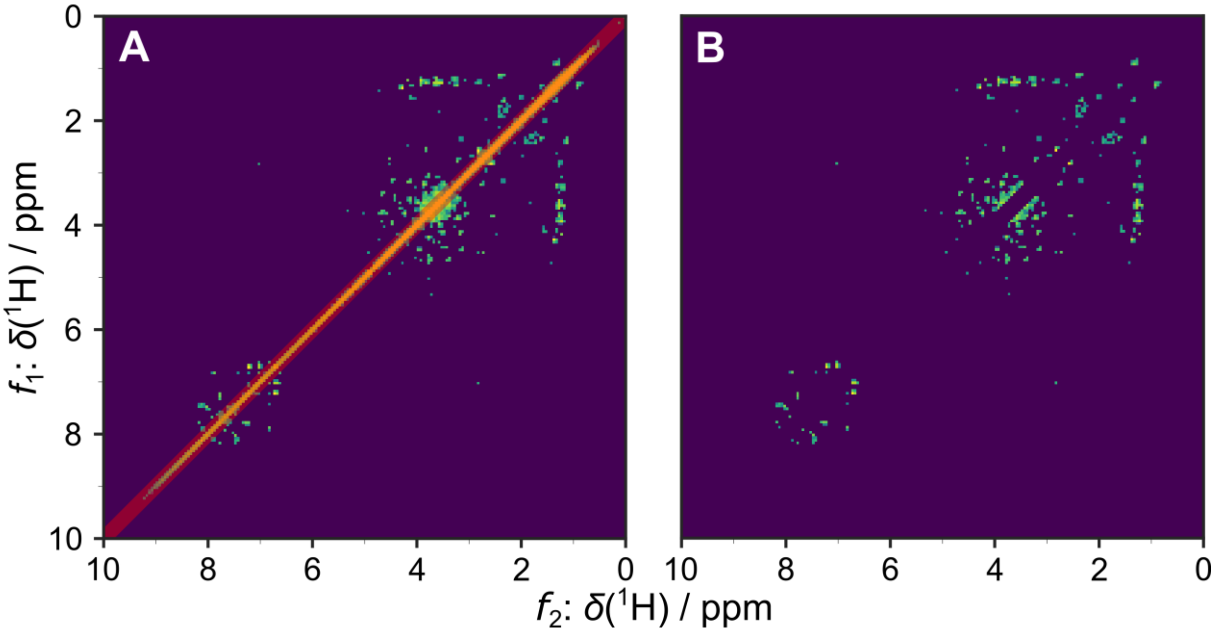

Figure S13 Binned ^{1}H,^{1}H COSY spectrum of the seawater sample with (A) and without (B) diagonal signals. Signals inside or on the edge of the red shaded area of A were removed to produce the spectrum displayed in panel B.

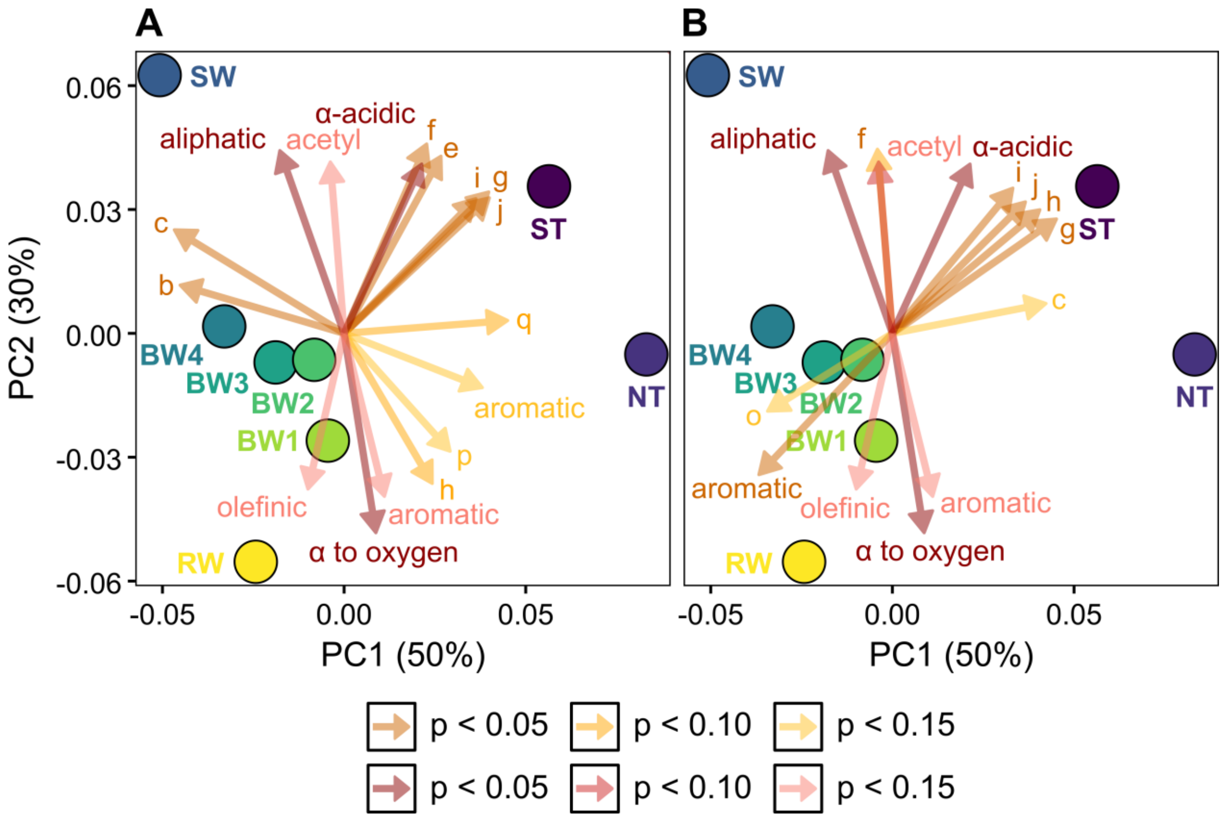

Figure S14 Principal coordinate analysis (PCoA) based on the Bray-Curtis dissimilarity matrix of binned $^1H,^1H$ COSY NMR data. Correlations with relative abundances of structural feature groups derived from 1D ^1H NMR (red vectors) and 2D COSY NMR (ocher vectors) without (A) and with (B) removal of the diagonal signals were fitted *post hoc* to the PCoA data. Please note that the ocher vector representing aromatic structural motifs derived from 2D COSY reflects the combined contributions of all aromatic regions. Correlations with $p < 0.10$ (standard colors) and with $p < 0.05$ (dark colors) are shown. Occasionally, correlations with $p < 0.15$ are displayed (light colors). The projections of sampling points onto vectors have maximum correlation with the respective variables.

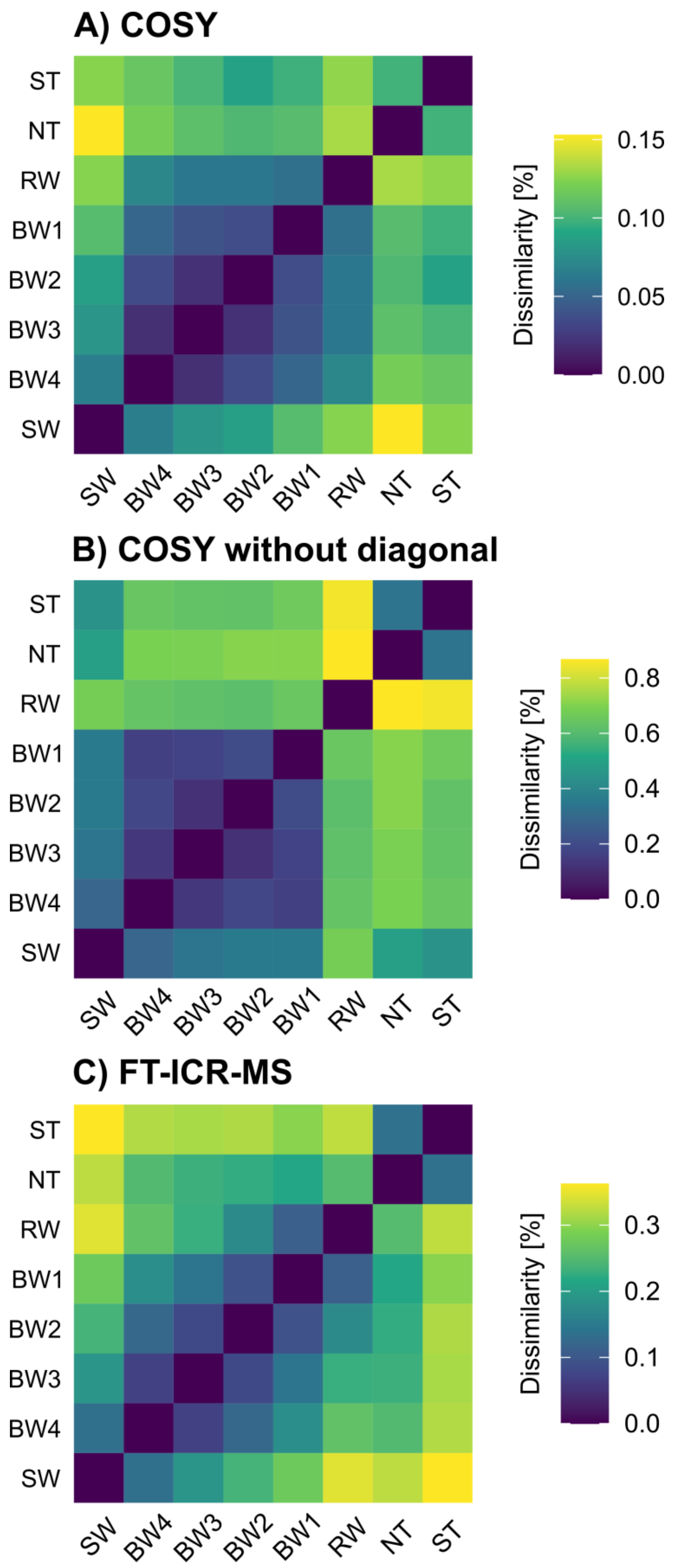

Figure S15 Heatmaps of Bray-Curtis dissimilarity matrix based on A) binned $^{1}H,^{1}H$ COSY NMR data, B) binned $^{1}H,^{1}H$ COSY NMR data after removing the diagonal signals and C) FT-ICR-MS data.

S2.4 Comparison of diagonal and cross-peak intensities

As summarized in Table S6, the intensity of the cross-peaks in the analyzed samples accounted for only 0.29 to 2.23% of the total spectral intensity. The average signal intensity of diagonal peaks was approximately 28 to 40 times greater than that of the cross-peaks, which falls within the expected dynamic range for $^{1}H,^{1}H$ COSY NMR spectra. Notably, the largest ratios of average normalized signal intensity for diagonal peaks to cross-peaks were observed in the low tide samples, with values of ~35 and ~40, respectively. Therefore, we rule out a dynamic range issue as a general explanation for the lower number of cross-peaks in the river water sample. The two most likely explanations for this observation are consequently: a) the river water sample is structurally less complex, or b) the river water sample contains a few dominant structural features that produced the observed cross-peaks, while the majority of compounds had structurally diverse motifs, resulting in weaker cross-peaks that fell below the detection limit. As discussed in more detail in the main manuscript, we consider the second explanation unlikely.

Table S6 Comparison of diagonal and cross-peak intensities for a bin size of 0.05 ppm. Values are given for the COSY spectra processed as described in S1.6.

**Sample**	**Cross-peaks**			**Diagonal peaks**		
	**I* [%]**	$\mathbf{N_{bins}^{+}}$	$\mathbf{\bar{I}^{\#}}$	**I* [%]**	$\mathbf{N_{bins}^{+}}$	$\mathbf{\bar{I}^{\#}}$
Seawater (**SW**)	1.66	469	3.55E-03	98.34	833	1.18E-01
Brackish water (**BW4**)	0.92	231	3.98E-03	99.08	795	1.25E-01
Brackish water (**BW3**)	0.69	197	3.53E-03	99.31	796	1.25E-01
Brackish water (**BW2**)	0.70	207	3.37E-03	99.30	792	1.25E-01
Brackish water (**BW1**)	0.92	198	4.62E-03	99.08	767	1.29E-01
River water (**RW**)	0.29	52	5.62E-03	99.71	572	1.74E-01
Neap tide (**NT**)	2.23	782	2.86E-03	97.77	972	1.01E-01
Spring tide (**ST**)	2.04	873	2.34E-03	97.96	1052	9.31E-02

*total intensity of cross- or diagonal peak bins, respectively.
+number of cross- or diagonal peak bins with an intensity signal, respectively.
#average signal intensity in cross- or diagonal peak bins with an intensity signal, respectively.

S2.5 Spearman correlation of COSY section integrals, MS compound groups, and MS derived molecular parameters

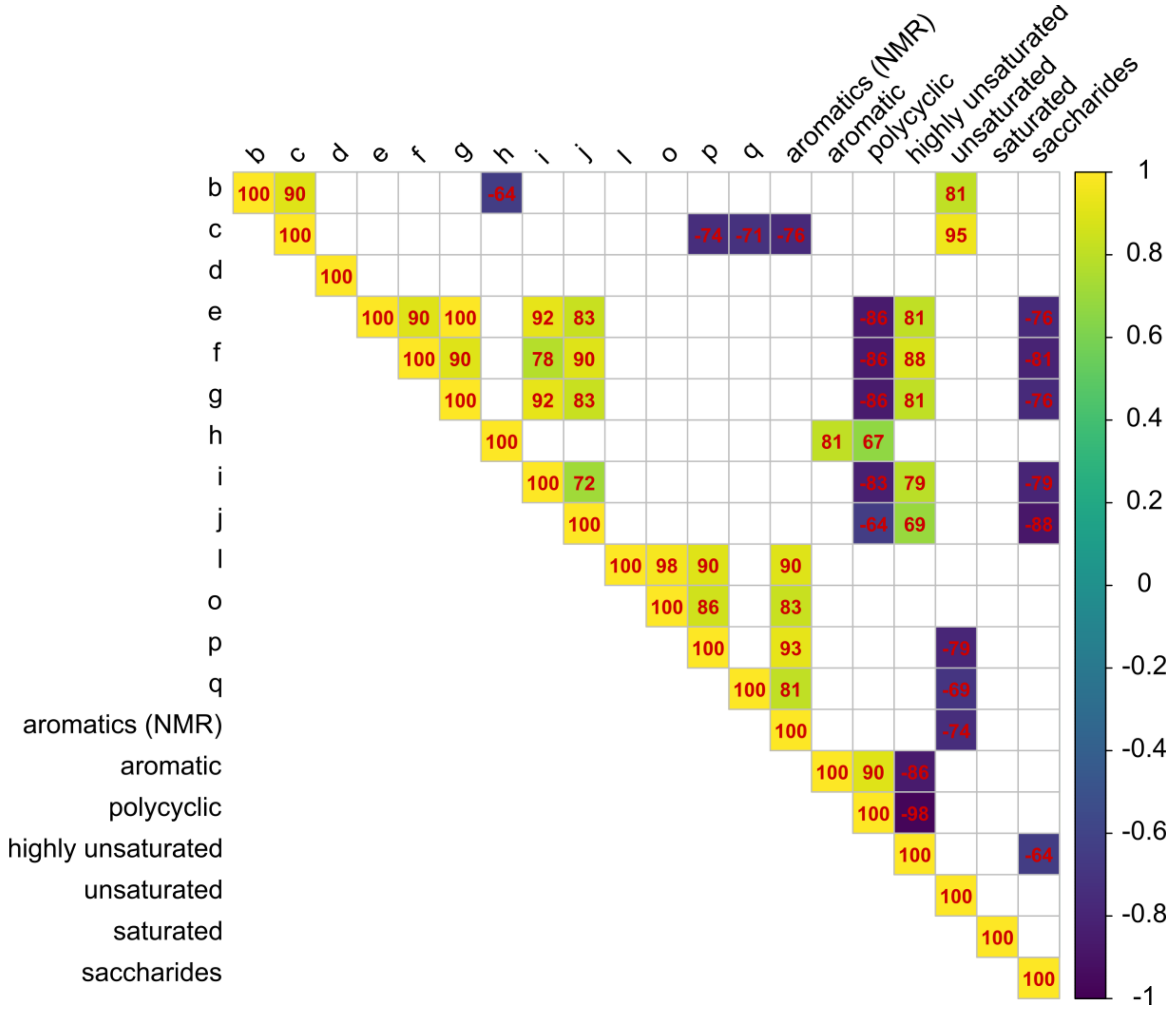

Figure S16 Significant Spearman correlations of selected COSY section integrals with MS compound groups ($p \leq 0.10$) colored according to their correlation coefficients. Coefficients are given in percentages and were calculated including all eight samples.

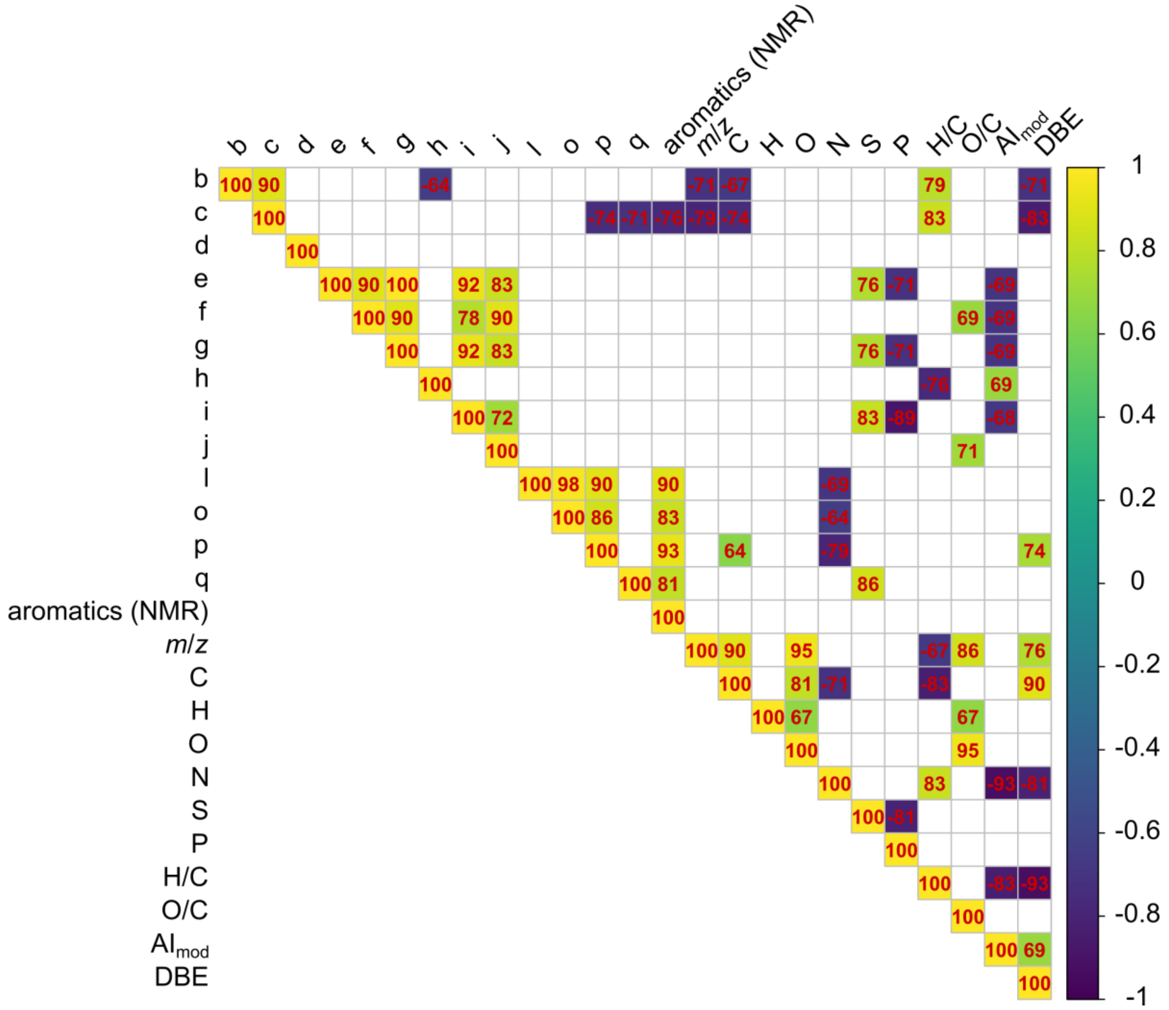

Figure S17 Significant Spearman correlations of COSY section integrals with MS derived molecular parameters ($p \leq 0.10$) colored according to their correlation coefficients. Coefficients are given in percentages and were calculated including all eight samples.

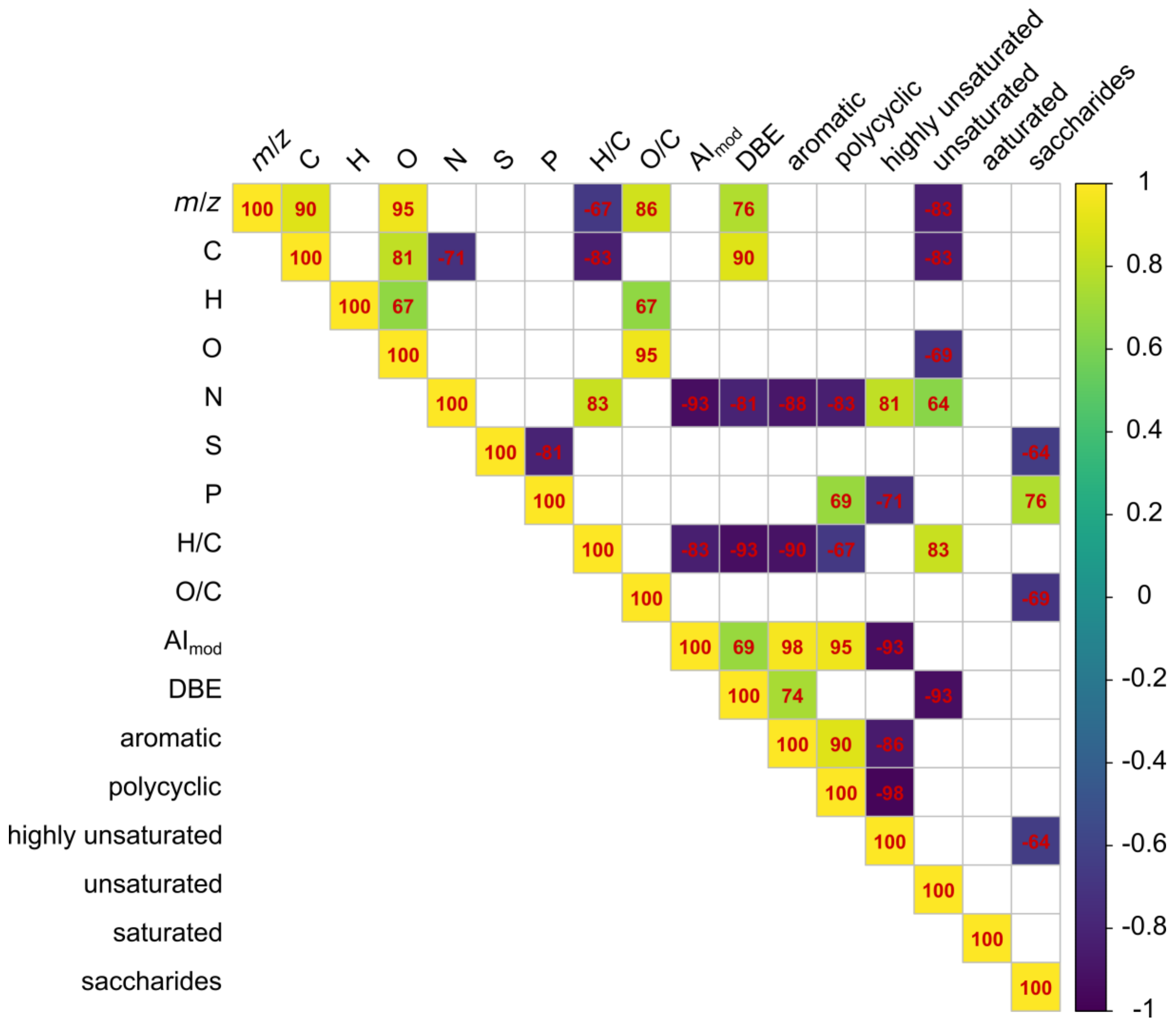

Figure S18 Significant Spearman correlations of MS compound groups with MS derived molecular parameters ($p \leq 0.10$) colored according to their correlation coefficients. Coefficients are given in percentages and were calculated including all eight samples.

S2.6 Detection of saccharides by MS and NMR

Saccharides can occur as glycosides. An example is quercetin 3-*O*-α-L-arabinopyranoside (**1**), which has been isolated from *Rhizophora mangle* (Figure S19).[9] This compound has the molecular formula $C_{20}H_{18}O_{11}$, associated with H/C and O/C values of 0.9 and 0.55, respectively. Thus, it is not detected as a saccharide using common MS compound group definitions. However, by NMR it would be partially detected as saccharide since six of the eleven non-exchangeable protons would display signals in NMR sections being indicative for saccharides (i.e., h and j in Table S4).

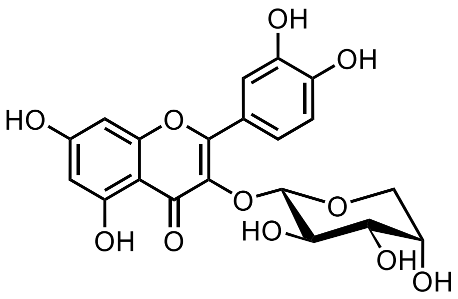

1

Figure S19 Structure of quercetin 3-*O*-α-L-arabinopyranoside (**1**), a flavonoid glycoside that has been isolated from *Rhizophora mangle*.[9]

S2.7 FT-ICR-MS-based relative abundance of sulfur-containing groups

Table S7 Relative abundance (in %) of sulfur-containing molecular formulas for the three distinct sulfur-containing groups along the transect and the total relative intensity of all sulfur-containing molecular formulas.

**Compound class**	**Dissolved black sulfur**	**Highly unsaturated**	**Aliphatic**	**Total S-containing**
**H/C**	< 0.6	> 0.6, < 1.0	> 1.0	
**O/C**	< 0.3	< 0.3	> 0.4	
Seawater (**SW**)	0.10	2.64	2.39	6.32
Brackish water (**BW4**)	0.48	2.34	2.77	7.99
Brackish water (**BW3**)	0.62	2.14	2.72	8.36
Brackish water (**BW2**)	0.86	2.20	2.33	8.29
Brackish water (**BW1**)	0.66	1.33	1.77	5.89
River water (**RW**)	0.93	1.34	1.18	5.60
Neap tide (**NT**)	0.56	1.42	5.66	16.32
Spring tide (**ST**)	0.11	0.51	7.23	16.49

S2.8 Prediction of NMR spectra resembling characteristic COSY NMR signals detected by CCorA

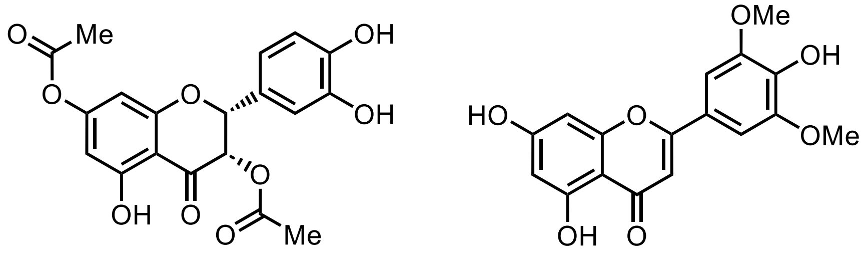

2 **3**

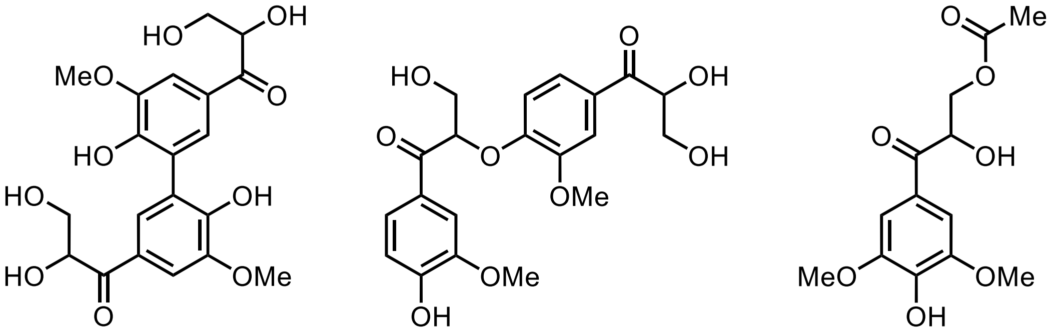

4 **5** **6**

Figure S20 Flavonoids[10,11] (**2** and **3**) and oxidized and/or acetylated[12] lignin degradation products (**4–6**) as potential compounds of the second source identified by CCorA. Lignin degradation products with β-aryl ether and biphenyl linkage were chosen since these linkages are most common in lignin.[13] The molecular formula of all compounds were also identified by FT-ICR-MS in at least one sample.

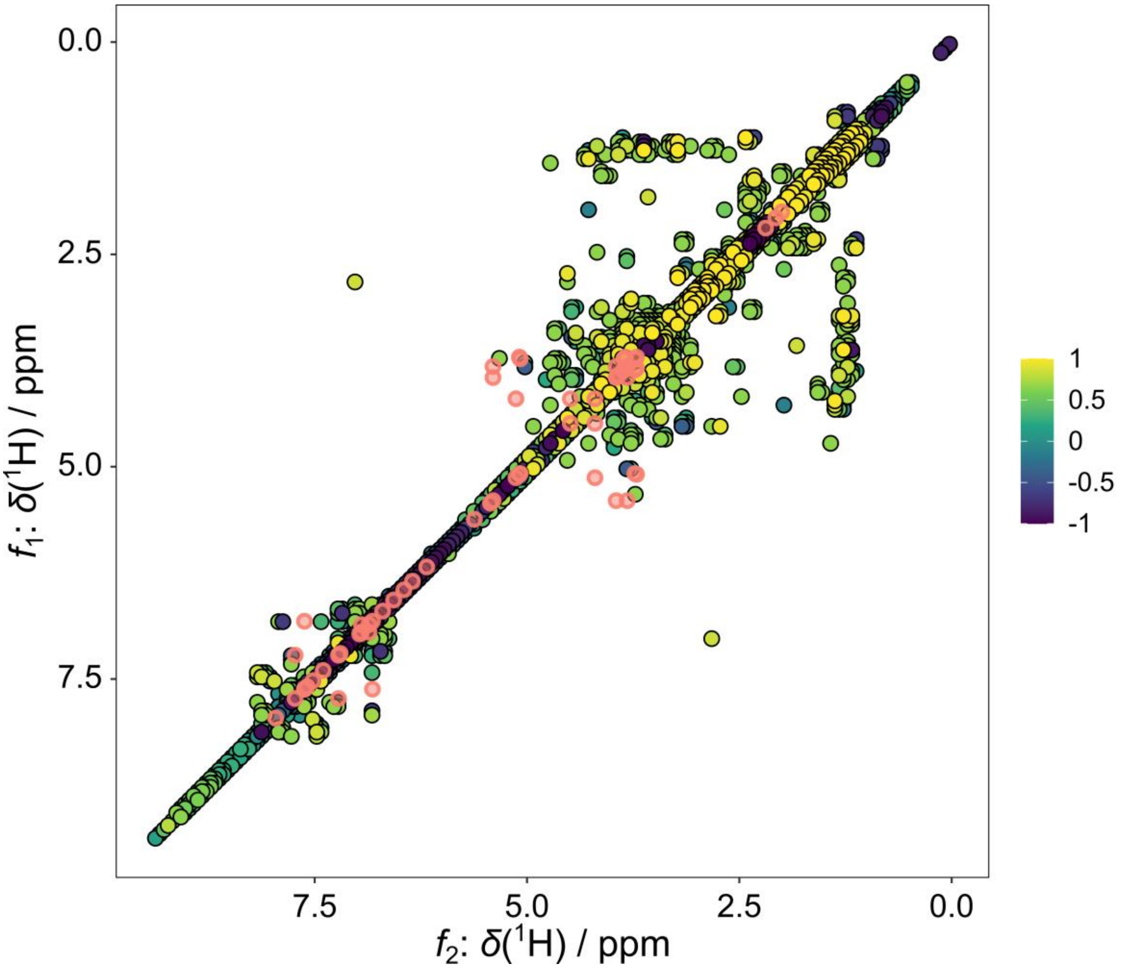

Figure S21 Canonical correlation analysis (CCorA) of the six river-to-ocean transect samples using the first two PCs of the PCoA based on NMR and MS data, respectively. Displayed are the color-coded correlations of the first canonical axis with the relative intensities of NMR data in a simplified NMR spectrum and overlayed with the predicted COSY NMR spectra of tannin- and lignin-derived compounds (coral dots, Figure S20). All correlations between the first canonical axis and the relative intensities of the NMR data are shown without applying any significance filtering. The significance of canonical correlations was calculated as permutational probability associated with Pillai's trace statistics with 719 permutations (p = 0.04).

S3 Supplementary References

(1) Knoke, M.; Dittmar, T.; Zielinski, O.; Kida, M.; Asp, N. E.; de Rezende, C. E.; Schnetger, B.; Seidel, M. Outwelling of Reduced Porewater Drives the Biogeochemistry of Dissolved Organic Matter and Trace Metals in a Major Mangrove-Fringed Estuary in Amazonia. *Limnol. Oceanogr.* **2024**, *69* (2), 262–278. https://doi.org/10.1002/lno.12473.

(2) Merder, J.; Freund, J. A.; Feudel, U.; Hansen, C. T.; Hawkes, J. A.; Jacob, B.; Klaproth, K.; Niggemann, J.; Noriega-Ortega, B. E.; Osterholz, H.; Rossel, P. E.; Seidel, M.; Singer, G.; Stubbins, A.; Waska, H.; Dittmar, T. ICBM-OCEAN: Processing Ultrahigh-Resolution Mass Spectrometry Data of Complex Molecular Mixtures. *Anal. Chem.* **2020**, *92* (10), 6832–6838. https://doi.org/10.1021/acs.analchem.9b05659.

(3) Rivas-Ubach, A.; Liu, Y.; Bianchi, T. S.; Tolić, N.; Jansson, C.; Paša-Tolić, L. Moving beyond the van Krevelen Diagram: A New Stoichiometric Approach for Compound Classification in Organisms. *Anal. Chem.* **2018**, *90* (10), 6152–6160. https://doi.org/10.1021/acs.analchem.8b00529.

(4) Wu, X.; Wu, L.; Liu, Y.; Zhang, P.; Li, Q.; Zhou, J.; Hess, N. J.; Hazen, T. C.; Yang, W.; Chakraborty, R. Microbial Interactions with Dissolved Organic Matter Drive Carbon Dynamics and Community Succession. *Front. Microbiol.* **2018**, *9*, 1234. https://doi.org/10.3389/fmicb.2018.01234.

(5) Koch, B. P.; Dittmar, T. From Mass to Structure: An Aromaticity Index for High-Resolution Mass Data of Natural Organic Matter. *Rapid Commun. Mass Spectrom.* **2006**, *20* (5), 926–932. https://doi.org/10.1002/rcm.2386.

(6) Koch, B. P.; Dittmar, T. Erratum: From Mass to Structure: An Aromaticity Index for High-Resolution Mass Data of Natural Organic Matter (Rapid Communications in Mass Spectrometry (2006) 20 (926–932) DOI: 10.1002/rcm.2386). *Rapid Commun. Mass Spectrom.* **2016**, *30* (1), 250. https://doi.org/10.1002/rcm.7433.

(7) Zsolnay, A.; Baigar, E.; Jimenez, M.; Steinweg, B.; Saccomandi, F. Differentiating with Fluorescence Spectroscopy the Sources of Dissolved Organic Matter in Soils Subjected to Drying. *Chemosphere* **1999**, *38* (1), 45–50. https://doi.org/10.1016/S0045-6535(98)00166-0.

(8) Huguet, A.; Vacher, L.; Relexans, S.; Saubusse, S.; Froidefond, J. M.; Parlanti, E. Properties of Fluorescent Dissolved Organic Matter in the Gironde Estuary. *Org. Geochem.* **2009**, *40* (6), 706–719. https://doi.org/10.1016/j.orggeochem.2009.03.002.

(9) Kandil, F. E.; Grace, M. H.; Seigler, D. S.; Cheeseman, J. M. Polyphenolics in Rhizophora Mangle L. Leaves and Their Changes during Leaf Development and Senescence. *Trees - Struct. Funct.* **2004**, *18* (5), 518–528. https://doi.org/10.1007/s00468-004-0337-8.

(10) Li, D. L.; Li, X. M.; Peng, Z. Y.; Wang, B. G. Flavanol Derivatives from Rhizophora Stylosa and Their DPPH Radical Scavenging Activity. *Molecules* **2007**, *12* (5), 1163–1169. https://doi.org/10.3390/12051163.

(11) Nebula, M.; Harisankar, H. S.; Chandramohanakumar, N. Metabolites and Bioactivities of Rhizophoraceae Mangroves. *Nat. Prod. Bioprospect.* **2013**, *3* (5), 207–232. https://doi.org/10.1007/s13659-013-0012-0.

(12) Del Río, J. C.; Marques, G.; Rencoret, J.; Martínez, Á. T.; Gutiérrez, A. Occurrence of Naturally Acetylated Lignin Units. *J. Agric. Food Chem.* **2007**, *55* (14), 5461–5468. https://doi.org/10.1021/jf0705264.

(13) Bugg, T. D. H.; Ahmad, M.; Hardiman, E. M.; Rahmanpour, R. Pathways for Degradation of Lignin in Bacteria and Fungi. *Nat. Prod. Rep.* **2011**, *28* (12), 1883–1896. https://doi.org/10.1039/c1np00042j.
